# Conscious awareness, sensory integration, and evidence accumulation in bodily self-perception

**DOI:** 10.1073/pnas.2503629122

**Published:** 2025-12-03

**Authors:** Renzo C. Lanfranco, Sucharit Katyal, August Hägerdal, Xiaole Luan, Victoria Nos, H. Henrik Ehrsson

**Affiliations:** ^a^Department of Neuroscience, Karolinska Institutet, Stockholm 171 65, Sweden; ^b^Department of Clinical Neuroscience, Karolinska Institutet, Stockholm 171 65, Sweden; ^c^Max Planck UCL Centre for Computational Psychiatry and Ageing Research, University College London, London WC1B 5EH, United Kingdom; ^d^Department of Psychology, University of Copenhagen, Copenhagen 1353, Denmark; ^e^Faculty of Life Sciences, Reutlingen University, Reutlingen 72762, Germany

**Keywords:** self-awareness, body ownership, consciousness, self, multisensory processing

## Abstract

Conscious awareness refers to the subjective experience of perceiving, thinking, and feeling and the ability to report these experiences. These perceptions and thoughts are experienced as bound to an individual self. A fundamental aspect of this self-consciousness is the sense of bodily self—the experience of one’s physical presence distinct from the external world, serving as the spatial reference point for conscious perceptions. A key component of the bodily self is body ownership, the experience of the body as one’s own. Research shows this sense involves integrating signals from different sensory modalities, including vision, touch, and proprioception, into a coherent multisensory percept. However, the relationship between body ownership and conscious awareness remains unclear. To investigate this, we developed a psychophysical paradigm to objectively quantify multisensory integration, conscious awareness, and their relationships within a bodily illusion manipulating body ownership perception. Using signal-detection analysis, metacognitive computational modeling, and drift–diffusion modeling, we found conscious awareness reports closely matched objective discrimination of body ownership. This relationship remained consistent across different levels of multisensory integration and evidence accumulation. A visuotactile control experiment revealed that this strong conscious access is specific to body ownership, not general to multisensory integration. These findings suggest that conscious awareness has continuous and prioritized access to body ownership, implying that the self-related form of multisensory integration supporting body ownership is largely implemented at the level of conscious processing. This provides theoretical insight into how conscious awareness and the bodily self are intertwined, with wide-reaching implications for consciousness and body representation research.

## Significance

How is conscious awareness related to our sense of bodily self? Researchers have long believed that many sensory signals from one’s body are processed without reaching conscious awareness, with studies on consciousness mainly focusing on how we become aware of visual and auditory information after it has first been processed subconsciously. Using bodily illusion experiments and computational modeling, we show that bodily self-perception is surprisingly closely connected to conscious awareness, shedding light on this important connection.

Humans, and likely other mammals, develop, as they mature, a unitary sense of self, where thoughts, actions, and sensations are coherently bound together. This sense of self is a key aspect of human consciousness, anchoring the body as the reference point from which perceptions of oneself and the world emerge. We perceive our bodies as distinct, unitary spatial entities separate from the external environment. As psychologist William James observed, the sense of self is deeply intertwined with bodily experiences; the continuous presence of the body in our consciousness forms a constant and fundamental backdrop for all other experiences (1). A crucial element of this bodily self is the sense of body ownership—the perceptual experience of the body as one’s own. Twenty-five years of research have shown that the sense of body ownership arises from the integration of visual, tactile, proprioceptive, and other sensory signals, which the brain infers to share a common origin (2, 3) based on past experiences and a stored central body representation (4–7). However, despite body ownership being foundational to self-consciousness, the relationship between body ownership and conscious awareness remains poorly understood.

In cognitive neuroscience and psychological science, conscious awareness typically refers to the subjective experience of perceiving, thinking, and feeling, along with the ability to report these experiences. It involves a state in which an individual is aware of sensory inputs and able to describe them. Unconscious perception, on the other hand, refers to the processing of sensory information that influences behavior without being accompanied by subjective experience. These concepts are fundamental to most leading neuroscience-inspired models of conscious awareness and are supported by a large body of empirical work showing that visual and auditory information can be processed to some extent without awareness (8–18). One particularly influential theory, the global neuronal workspace theory (GNWT), posits that when a stimulus surpasses a threshold for conscious processing, information pertaining to it is ‘broadcast’ throughout the brain. The characteristics of a stimulus and the subject’s state of attention can influence this process, thereby affecting the sensory input’s accessibility to awareness (19, 20). This theory also delineates different levels of nonconscious processing (21, 22), such as when the bottom–up sensory signal is so weak that it fails to “ignite” the neural networks of awareness (subliminal processing) or when the sensory signal is strong enough but is still not ‘broadcast’ into awareness owing to a lack of top–down attentional amplification (preconscious processing). However, these concepts primarily stem from studies on visual perception, and it is unclear how they extend to the more complex sensory integration that underpins the sense of body ownership and the bodily self. Some studies have compared conscious and unconscious processing of stimuli related to self-concept and visual self-recognition. For example, several studies have shown that self-related visual information, such as one’s face (23–26) and one’s name (27, 28), is prioritized for access to awareness over information about other individuals. However, these studies have used unisensory stimuli and have investigated conceptual and semantic representations of self (self-concept), rather than the immediate multisensory perceptual experience of oneself that is central to body ownership and the bodily self.

The sense of body ownership can be studied experimentally in healthy participants using bodily illusions (29), with the most commonly used being the rubber hand illusion (30). In the RHI, the participant feels that a rubber hand in full view is their own hand; this illusion is elicited by repeatedly stroking the fake hand alongside the participant’s real hand, which is hidden. For this feeling of illusory rubber hand ownership to arise, certain perceptual rules must be met: the rubber hand and real hand must be stroked synchronously (temporal rule) and placed in similar positions and orientations (spatial rule), and the rubber hand must resemble a human hand (31–33). These rules mirror the principles of multisensory integration, i.e., that signals from different senses that correspond well in space and time and other stimulus properties are combined into single perceptual events and objects (2, 34). The importance of visuotactile congruence in driving the RHI has been particularly well researched. We know that even small variations in the degree of asynchrony modulate body ownership sensations, with smaller asynchrony leading to a stronger RHI (2, 35, 36). Such asynchrony-related body ownership modulation is also reflected in changes in neural activity in multisensory brain regions (5, 37, 38), further underlining the link between multisensory integration and body ownership.

Body ownership clearly involves conscious perception, as the RHI is vivid and can be described by most participants; it is typically recorded by having participants fill out a questionnaire (30, 39, 40). However, this does not mean that unconscious processes are not also involved or that they do not make a significant contribution. Indeed, a few studies have investigated how unconscious multisensory interactions can influence visual awareness and various indirect body ownership measures. It has been shown that the illusory sensation of ownership over a fake hand during the RHI boosts visual conscious processing in a binocular rivalry task (41). In a similar vein, continuous flash suppression experiments have revealed that visually suppressed stimuli break suppression and become visible faster when combined with congruent tactile (42) or proprioceptive (43) stimuli than when combined with incongruent stimuli in bodily illusion paradigms. It has also been reported that unconscious visual signals rendered invisible through continuous flash suppression influence indirect behavioral measures of a full-body illusion when paired with congruent consciously perceivable tactile stimuli (44). These studies support the unconscious processing of multisensory signals, echoing the common assumption that some bodily processing occurs outside awareness. This view dates back at least to the early conceptualization of a “body schema”: a representation of the body’s position in space used for action, believed to be constructed at least partially unconsciously (45–48). However, rigorous methods for objectively quantifying conscious versus unconscious processes in body ownership perception are lacking, so the relationship between the two remains unclear.

Here, we take advantage of recent advances in psychophysical approaches to the RHI (35, 36, 49, 50) and computational modeling of perceptual awareness (51, 52) and evidence accumulation (53, 54) to investigate conscious and unconscious processing in body ownership perception. We developed a psychophysical RHI paradigm with two rubber hands, where participants were required to choose which hand felt more like their own in a forced-choice procedure. In this setup, the visuotactile asynchronies between the felt (but unseen) touches on the real hand and the seen touches on the rubber hands were systematically varied on a trial-by-trial basis to test the effects of visuotactile asynchrony on body ownership discrimination and the vividness of subjective body ownership experience. Using signal detection theory (SDT) and computational modeling of metacognition, we quantified the sensitivity of body ownership discriminations to visuotactile asynchronies (Type-1 sensitivity, or “body ownership sensitivity”), the sensitivity of perceptual awareness reports to perceptual discriminations (Type-2 sensitivity, or “perceptual awareness sensitivity”), and the degree to which perceptual awareness reports accurately reflected body ownership information (perceptual awareness efficiency; the ratio of Type-2 sensitivity to Type-1 sensitivity) across different levels of visuotactile asynchrony (i.e., varying degrees of multisensory integration). This approach enabled us to investigate the relationship between conscious awareness and objective body ownership perception, testing the hypothesis that body ownership discrimination is more sensitive to shorter asynchronies than perceptual awareness judgments are.

Contrary to our expectations, the results revealed a very close relationship between body ownership sensitivity and perceptual awareness sensitivity, with awareness reports reliably reflecting objective body ownership perception, regardless of visuotactile asynchrony level. Expanding on this finding, in two additional experiments, we examined whether this close relationship also holds when the RHI emerges and gradually strengthens through repeated visuotactile stimulation, driven by the accumulation of multisensory evidence over time (55, 56). We found that the close relationship between body ownership sensitivity and perceptual awareness sensitivity persisted, regardless of the number of visuotactile stimuli used to induce the RHI. Moreover, using drift–diffusion modeling (DDM) in a speeded response-time task, we also observed that the rates of evidence accumulation for body ownership discrimination and awareness reports did not differ, underscoring this strong relationship. Taken together, our findings are theoretically significant because they suggest that consciousness has continuous and prioritized access to body ownership information. This indicates that the multisensory integration processes underlying this fundamental aspect of the bodily self are highly dependent on conscious awareness. This conclusion challenges prevailing views in the conscious awareness and body representation literature, which typically assume that unconscious processes contribute to perception, including bodily self-perception. Instead, our results indicate that conscious awareness and the bodily self are deeply intertwined.

## Results

### Objective Quantification of Body Ownership and Conscious Access.

**Fig. 1.**
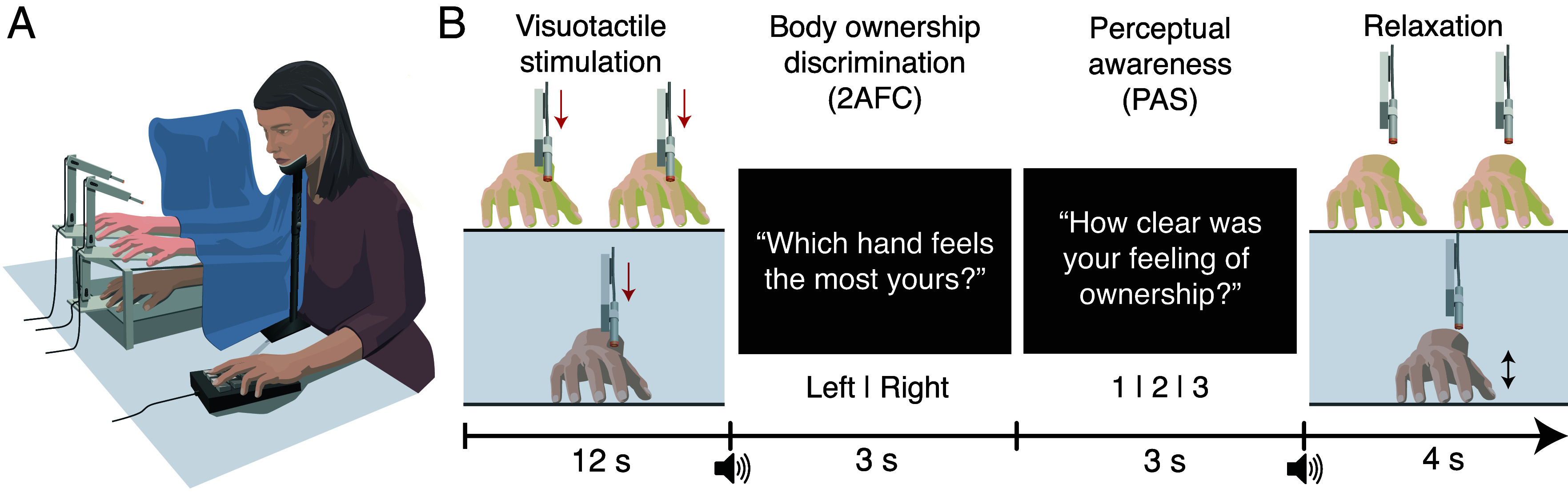
(*A*) Experimental setup. Two robotic arms apply touches to both rubber hands placed on top of the upper platform, and one robotic arm applies touches to the participant’s real hand under this platform, on a lower level. (*B*) Trial schematics. The robotic arms tap the rubber hands and real hand with different degrees of asynchrony between the rubber hands and the real hand; one rubber hand is always synchronously tapped with the real hand one (randomized for each trial), which is the condition that produces the strongest RHI, and the other rubber hand is tapped at one of five levels of asynchrony (from 18 to 150 ms). Next, an auditory cue informs participants to respond using a numeric keypad to the following: Which rubber hand feels most like their own (2AFC; left or right, using left or right arrows, respectively), and what is the clarity of their subjective body ownership experience for the rubber hand chosen (PAS; 3-point scale, using keys 1, 2, or 3). Afterward, the participants wiggled their fingers several times to break the illusion and reduce carry-over effects and relaxed their fingers for the remainder of the rest period. Finally, an auditory cue informs them when the next trial begins. A chin rest minimizes head movement.

In Experiment 1, we induced the RHI simultaneously with two rubber hands using a robotic setup (Fig. 1*A*; see *Materials and Methods* for more details). Each robot arm delivered a series of six touches on the participant’s hand and the rubber hands during a 12-s period. We manipulated the stimulation asynchrony between the felt touch (on the real but hidden hand) and the seen touch (on the fake but visible rubber hands), with one rubber hand being tapped synchronously with the real hand and the other rubber hand being tapped asynchronously per trial. After stimulation, the participants completed a body ownership discrimination task where they had to report which of the two rubber hands felt most like theirs. The participants subsequently rated the clarity of their body ownership experience using a Perceptual Awareness Scale (PAS), where 1 means “unclear,” 2 means “vague,” and 3 “clear,” on the basis of the clarity of their ownership experience for the chosen rubber hand (Fig. 1*B*). This RHI paradigm allowed us to quantify the sensitivity of body ownership to visuotactile signals (i.e., the extent to which body ownership discriminates visuotactile asynchronies, d′; see *Analysis*), and the access of conscious awareness to body ownership sensitivity (i.e., the extent to which the clarity of subjective experience predicts body ownership discrimination performance, meta-d′; see *Analysis*) by using SDT analyses and computational modeling of metacognition.

### The Sense of Body Ownership Is Sensitive to Asynchronies in Visuotactile Stimulation Starting from ≈30 ms.

**Fig. 2.**
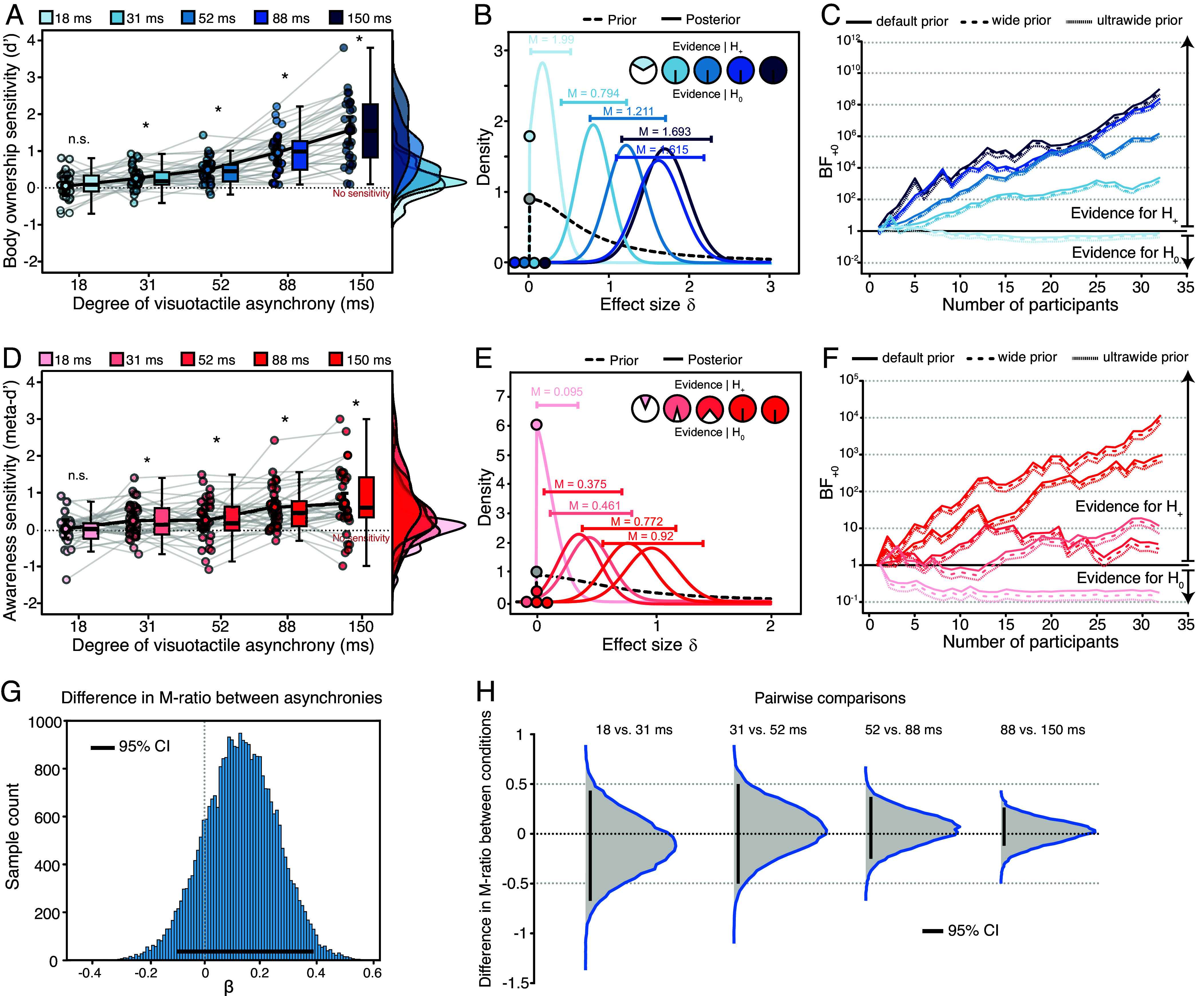
Body ownership, perceptual awareness sensitivity, and perceptual awareness efficiency results. (*A*–*C*) Body ownership sensitivity. (*A*) Individual one-sample *t* tests against zero. Body ownership d′ was significantly above zero at 31, 52, 88, and 150 ms of visuotactile asynchrony, but not at 18 ms. Bayesian one-sample *t* tests against zero (one-tailed). (*B*) Prior and posterior distributions. Bayes factors offered varying degrees of evidence in favor of different hypotheses across different levels of asynchrony. Specifically, the null hypothesis model received anecdotal support for 18 ms of asynchrony (i.e., body ownership d′ ≤ 0). In contrast, the alternative hypothesis model (i.e., body ownership d′ > 0) garnered overwhelming support for 31, 52, 88, and 150 ms of asynchrony. The estimated population effect sizes, medians, and 95% central credible intervals for each asynchrony condition are depicted. (*C*) Sequential analysis with robustness assessment. The evidence for the null hypothesis model is extremely stable across different prior distributions for 18 ms of asynchrony, ranging from 0.27 to 1.2 (r = 0.072). The evidence for the alternative hypothesis model is extremely stable across different prior distributions, ranging from 1,029 to 1,198 (r = 0.77) for 31 ms of asynchrony, ranging from 7.36 × 10^5^ to 7.44 × 10^5^ (r = 1.2) for 52 ms of asynchrony, ranging from 2.66 × 10^8^ to 2.67 × 10^8^ (r = 1.5) for 88-ms of asynchrony, and ranging from 7.58 × 10^8^ to 7.65 × 10^8^ (r = 1.5) for 150-ms of asynchrony. (*D*–*F*) Perceptual awareness sensitivity calculated using the maximum likelihood estimation method. (*D*) Individual one-sample *t* tests against zero. Perceptual awareness d′ was significantly above zero with 31, 52, 88, and 150 ms of stimulation asynchrony, but not with 18 ms. Bayesian one-sample t tests against zero (one-sided). (*E*) Prior and posterior distributions. Bayes factors offered varying degrees of evidence in favor of different hypotheses across different levels of asynchrony. Specifically, the null hypothesis model received substantial support for 18-ms of asynchrony (i.e., perceptual awareness d′ ≤ 0). In contrast, the alternative hypothesis model (i.e., perceptual awareness d′ > 0) exhibited substantial support for 31 ms, strong support for 52 ms, and extreme support for 88 and 150 ms of asynchrony. The estimated population effect sizes, medians, and 95% central credible intervals for each asynchrony condition are shown. (*F*) Sequential analysis with robustness assessment. The evidence for the null hypothesis model is extremely stable across different prior distributions, ranging from 0.076 to 0.99 (r = 0.04 × 10^−5^) for 18 ms of asynchrony. The evidence for the alternative hypothesis model is extremely stable across different prior distributions, ranging from 6.8 to 11.6 (r = 0.39) for 31 ms of asynchrony, ranging from 2.18 to 4.53 (r = 0.29) for 52 ms of asynchrony, ranging from 7,684 to 8,368 (r = 0.91) for 88 ms of asynchrony, and ranging from 726 to 859 (r = 0.752) for 150 ms of asynchrony. (*G* and *H*) Perceptual awareness efficiency (M-ratio) assessed by a Bayesian hierarchical model. (*G*) Estimation of differences in M-ratio between different degrees of visuotactile asynchronies, assessed with 95% highest density interval (HDI), suggesting no difference between degrees of asynchrony conditions. Each histogram represents posterior densities of M-ratio for all conditions. The results show no difference (in log units) between condition posteriors. (*H*) Pairwise comparisons of posterior distributions between visuotactile stimulation asynchrony conditions provided no evidence for differences in perceptual awareness efficiency across degrees of visuotactile asynchrony. Error bars represent 95% credible intervals (CrI). Supplementary results for rubber hand bias and PAS reporting bias can be found in *SI Appendix*, Fig. S1 *A* and *B*, respectively.

The results from the RHI discrimination task revealed that body ownership sensitivity significantly increased across visuotactile asynchronies ranging from 18 to 150 ms (peatedeasures ANOVA; $\begin{array}{c} (F(4,\;124)= \end{array}$ $\begin{array}{c} 56.2,P<0.001,\;\eta p^{2}=0.644) \end{array}$ (Fig. 2*A*). Importantly, body ownership showed above-chance sensitivity at 31-ms of visuotactile asynchrony $\left(t\left(31\right)=4.79,\;P<0.001,\;d=0.85\right)$. but not at 18-ms of asynchrony $\left(t\left(31\right)=1.01,\;P=0.159,\;d=0.18\right)$, and Bayes factor analysis provided overwhelming evidence in favor of above-chance sensitivity from 30-ms of visuotactile asynchrony (all BF_+0_ > 100; see Fig. 2*B* for posterior distributions of each condition; equivalent results were obtained with different prior distributions, Fig. 2*C*), suggesting that the results are robust. Together, these results show that body ownership discrimination is highly sensitive to visuotactile asynchrony, with significant effects on sensitivity (d′) at asynchronies as short as approximately 30 ms, providing a strong foundation to test the hypothesis introduced earlier that body ownership awareness emerges only at longer delays.

### Perceptual Awareness Is Sensitive to Body Ownership Discrimination Starting from ≈30 ms of Visuotactile Asynchrony.

Strikingly, analysis of the perceptual awareness reports revealed that perceptual awareness sensitivity was significant from 31 ms of visuotactile asynchrony (Fig. 2*D*), closely mirroring the body ownership discrimination results reported above. We found that perceptual awareness sensitivity significantly increased across degrees of visuotactile asynchrony in the 18 to 150 ms range (repeated-measures ANOVA) $\begin{array}{c} \left(F(4,\;124)=10,P<0.001,\;\eta p^{2}=0.244\right) \end{array}$. Crucially, perceptual awareness sensitivity was above-chance from 31-ms of visuotactile asynchrony $\left(t\left(31\right)=2.82,\;P=0.004,\;d=0.5\right)$ but not at 18-ms of asynchrony $\begin{array}{c} \left(t\left(31\right)=-0.346,\;P=0.634,\;d=-0.06\right) \end{array}$, and Bayes factor analysis provided from strong to overwhelming evidence in favor of above-chance perceptual awareness sensitivity from 31-ms of asynchrony (all BF_+0_ > 10, except for the 52-ms condition which yielded BF_+0_ = 4.5; see Fig. 2*E* for posterior distributions of each condition; equivalent results were obtained with different prior distributions; see Fig. 2*F*), indicating that our results are robust. In summary, awareness reports appeared equally sensitive to visuotactile asynchrony information as objective discrimination, starting from ≈30 ms of asynchrony.

### The Proportion of Body Ownership Information that Reaches Conscious Awareness Is Constant Across Visuotactile Asynchronies.

Next, we investigated whether the amount of body ownership information that is accessed by perceptual awareness remains constant across the different levels of asynchrony, which would further underscore a tight and consistent relationship between perceptual awareness and body ownership. Using a Bayesian hierarchical model (52) adapted for multiple comparisons, we calculated perceptual awareness efficiency (M-ratio) across degrees of visuotactile asynchrony, which is an index capturing the relationship between body ownership sensitivity and perceptual awareness sensitivity (i.e., the proportion of available body ownership information that was accessed by perceptual awareness; see *Analysis* and *SI Appendix*, Fig. S1 *C* and *D*). The distribution of the posterior densities of M-ratio across all conditions did not differ, indicating that perceptual awareness efficiency was constant (Fig. 2*G*). Consistent with this, we found that all pairwise comparisons between asynchrony conditions (31 ms vs. 18 ms, 52 ms vs. 31 ms, and so on) encompassed zero, again indicating that perceptual awareness efficiency did not differ between degrees of asynchrony (Fig. 2*H*). A Bayesian one-way ANOVA on the single-subject M-ratio indices supported the observed null difference between asynchronies (*BF*_01_ = 6.199). In summary, this analysis revealed that as body ownership sensitivity increased with greater levels of asynchrony, perceptual awareness sensitivity increased proportionally, demonstrating that the proportion of body ownership information accessed by awareness remained constant.

### Control Experiment 1 Validates that the Psychophysical Findings Are Driven by Body Ownership Processing.

Earlier studies have shown that participants complete the current 2AFC-body ownership discrimination task on the basis of their body ownership perception rather than adopting an alternative strategy, such as judging visuotactile simultaneity (35, 36). To confirm that this held true in the current study, and also for the perceptual awareness reports, we added two control conditions conducted in a separate session. Control Experiment 1 replicated the setup, procedures, and task instructions of Experiment 1 described above except that we rotated the rubber hands 90 degrees clockwise, presenting them in an anatomically implausible orientation that abolishes the RHI (5, 31, 35, 36, 57). If participants exhibit no above-chance sensitivity with the rotated rubber hands, it implies that they followed instructions, using their sense of body ownership to perform the task as opposed to basing their reports on visuotactile simultaneity. As expected, we found that body ownership sensitivity was unaffected by the degree of asynchrony $\left(F(4,\;124)=0.03,P=0.99,\;\eta p2=0\right)$ and that it never departed from chance (all *P* > 0.329; all *BF*_+0_ < 0.27; see *SI Appendix*, Fig. S2 *A*–*C*). Notably, perceptual awareness reports across all degrees of visuotactile asynchrony conditions exhibited an overwhelming preference for “unclear feeling” (1 on the PAS; see *SI Appendix*, Fig. S2*D*), which was consistent with the absence of body ownership experience with rotated rubber hands. This finding confirms that participants followed task instructions and based their Type-1 and Type-2 reports on their feelings of illusory body ownership.

### Control Experiment 2 Confirms that Conscious Access Findings Are Specific to Body Ownership.

**Fig. 3.**
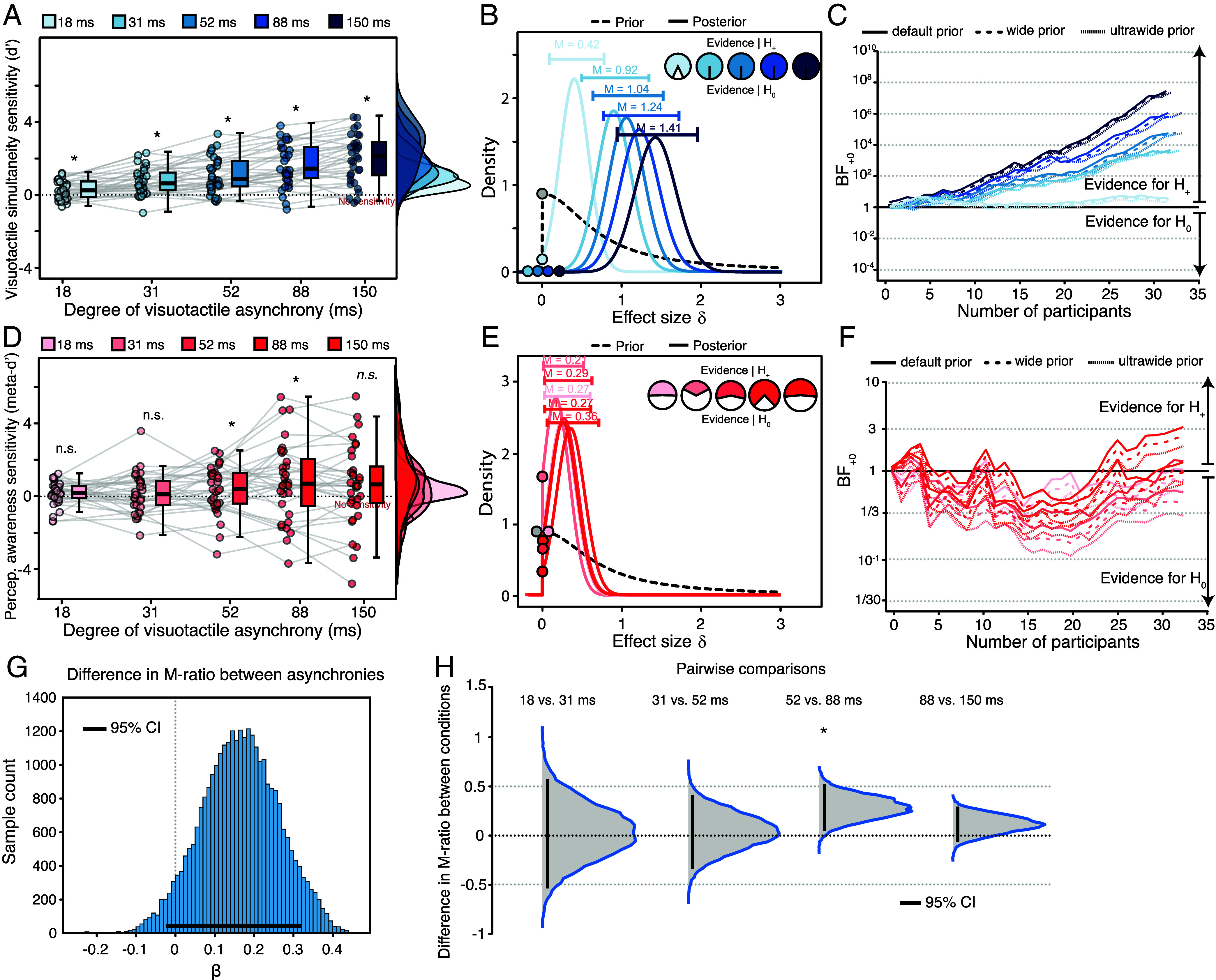
Visuotactile simultaneity, perceptual awareness sensitivity, and perceptual awareness efficiency results. (*A*–*C*) Visuotactile simultaneity sensitivity. (*A*) Individual one-sample *t* tests against zero. Visuotactile simultaneity d′ was significantly above zero at all degrees of asynchrony. Bayesian one-sample t-tests against zero (one-tailed). (*B*) Prior and posterior distributions. Bayes factors offered varying degrees of evidence in favor of the hypothesis model of above-chance sensitivity (i.e., body ownership d′ > 0). The estimated population effect sizes, medians, and 95% central credible intervals for each asynchrony condition are depicted. (*C*) Sequential analysis with robustness assessment. The evidence for the alternative hypothesis model is extremely stable across different prior distributions, ranging from 3.96 to 7.4 (r = 0.343) for 18 ms of asynchrony, ranging from 7,306 to 7,967 (r = 0.91) for 31 ms of asynchrony, ranging from 49,357– to 51,593 (r = 1.034) for 52 ms of asynchrony, ranging from 1.15 × 10^6^ to 1.16 × 10^6^ (r = 1.24) for 88 ms of asynchrony, and ranging from 1.378 × 10^7^ to 1.378 × 10^7^ (r = 1.41) for 150 ms of asynchrony. (*D*–*F*) Perceptual awareness sensitivity calculated using the maximum likelihood estimation method. (*D*) Individual one-sample *t* tests against zero. Perceptual awareness d′ was significantly above zero with 52 and 88 ms of visuotactile asynchrony, but not with 18, 31, and 150 ms. Note that Perceptual awareness d′ was marginally nonsignificant with 150 ms of asynchrony (*P* = 0.059). Bayesian one-sample *t* tests against zero (one-sided). (*E*) Prior and posterior distributions. Bayes factors offered varying degrees of evidence in favor of different hypotheses across different levels of asynchrony. Specifically, the null hypothesis model received anecdotal support for 18, 31, and 150 ms of asynchrony (i.e., perceptual awareness d′ ≤ 0), and substantial support for 88 ms of asynchrony. In contrast, the alternative hypothesis model (i.e., perceptual awareness d′ > 0) exhibited anecdotal support for 52 ms of asynchrony. The estimated population effect sizes, medians, and 95% central credible intervals for each asynchrony condition are shown. (*F*) Sequential analysis with robustness assessment. The evidence for the alternative hypothesis model is variable across different prior distributions, ranging from 0.59 to 1.78 (r = 0.16) for 18 ms of asynchrony, from 0.294 to 1.23 (r = 0.21) for 31 ms of asynchrony, from 0.77 to 2.1 (r = 0.17) for 52 ms of asynchrony, from 1.86 to 3.996 (r = 0.267) for 88 ms of asynchrony, and from 0.65 to 1.88 (r = 1.62) for 150 ms of asynchrony. (*G* and *H*) Perceptual awareness efficiency (M-ratio) assessed by a Bayesian hierarchical model. (*G*) Estimation of differences in M-ratio between different degrees of visuotactile asynchronies, assessed with 95% HDI, suggesting no difference between degrees of asynchrony conditions. Each histogram represents posterior densities of the M-ratio for all conditions. The results show no difference (in log units) between condition posteriors. (*H*) Pairwise comparison of posterior distributions between visuotactile stimulation asynchrony conditions indicated strong evidence for a difference in perceptual awareness efficiency only between 52 and 88 ms of visuotactile asynchrony. The posterior mean difference in M-ratio was higher for 88 ms than for 52 ms, with the 95% credible interval excluding zero. No other comparisons showed credible differences. Error bars represent 95% CrI.

The RHI emerges from the integration of visual, tactile, and proprioceptive signals. To confirm that the conscious access findings in Experiment 1 are specific to body ownership rather than reflecting a general feature of multisensory integration, we conducted a second control experiment. Control Experiment 2 replicated the setup and procedures of Experiment 1, with two key modifications: we replaced the rubber hands with two similarly sized blocks of wood to eliminate the RHI (32) and asked participants to perform a visuotactile simultaneity judgment task (which probes visuotactile integration) instead of a body ownership task. Thus, their task was to report the block that was tapped synchronously with their real hand and to rate the clarity of their visuotactile simultaneity experience. If body ownership information enjoys prioritized access to awareness compared to visuotactile processing in a nonbody ownership context, perceptual awareness efficiency should be higher for body ownership than for visuotactile simultaneity. We found that visuotactile simultaneity sensitivity increased across visuotactile asynchronies (*F* (2.12, 65.621) = 36.512, *P* < 0.001, *ηp* ^2^ = 0.541), showing above-chance sensitivity at all asynchronies $\left(\mathrm{all}\;t\left(31\right)>2.58,\;\mathrm{all}\;P<0.007\right)$ (Fig. 3*A*), and Bayes factor analysis provided moderate or overwhelming evidence in favor of above chance sensitivity at all asynchronies (BF_+0_ for 18 ms = 6.224, and all other BF_+0_ > 100; see Fig. 3*B* for posterior distributions; equivalent results were obtained with different prior distributions, Fig. 3*C*), suggesting that the results are robust. Perceptual awareness sensitivity did not vary significantly across visuotactile asynchronies $\begin{array}{c} \left(F(2.44,\;75.67)=2.125,P=0.116,\;\eta p^{2}=0.064\right) \end{array}$, showing above-chance sensitivity only at 52 (*t* (31) = 1.72, *P* = 0.048, (*d* = (0.303) and 88 ms of asynchrony (*t* (31) = 2.206, *P* = 0.017, (*d* = (0.183) (Fig. 3*D*), and Bayes factor analysis provided moderate or stronger evidence in favor of no above-chance sensitivity (all *BF*_+0_ < 0.54, except for *BF*_+0_ for 18 ms, which was 1.04; see Fig. 3 *E* and *F*). Perceptual awareness efficiency varied between 52 and 88 ms of visuotactile asynchrony, with a credibly higher M-ratio for the latter (Fig. 3 *G* and *H*). This contrasts with Experiment 1, where perceptual awareness efficiency showed no credible differences across visuotactile asynchronies. Importantly, perceptual awareness efficiency was significantly higher for body ownership (Experiment 1) than for visuotactile simultaneity (Control Experiment 2; *SI Appendix*, Fig. S3). A mixed repeated-measures ANOVA revealed a significant main effect of the experiment $\begin{array}{c} \left(F(1,\;62)=5.33,P=0.024,\;\eta p^{2}=0.079\right) \end{array}$. These findings indicate that the observed conscious access effect is specific to body ownership rather than a general feature of visuotactile integration.

### A Close Relationship Holds Between Body Ownership and Perceptual Awareness as the RHI Emerges Through Multisensory Evidence Accumulation Over Varying Numbers of Visuotactile Stimulations.

**Fig. 4.**
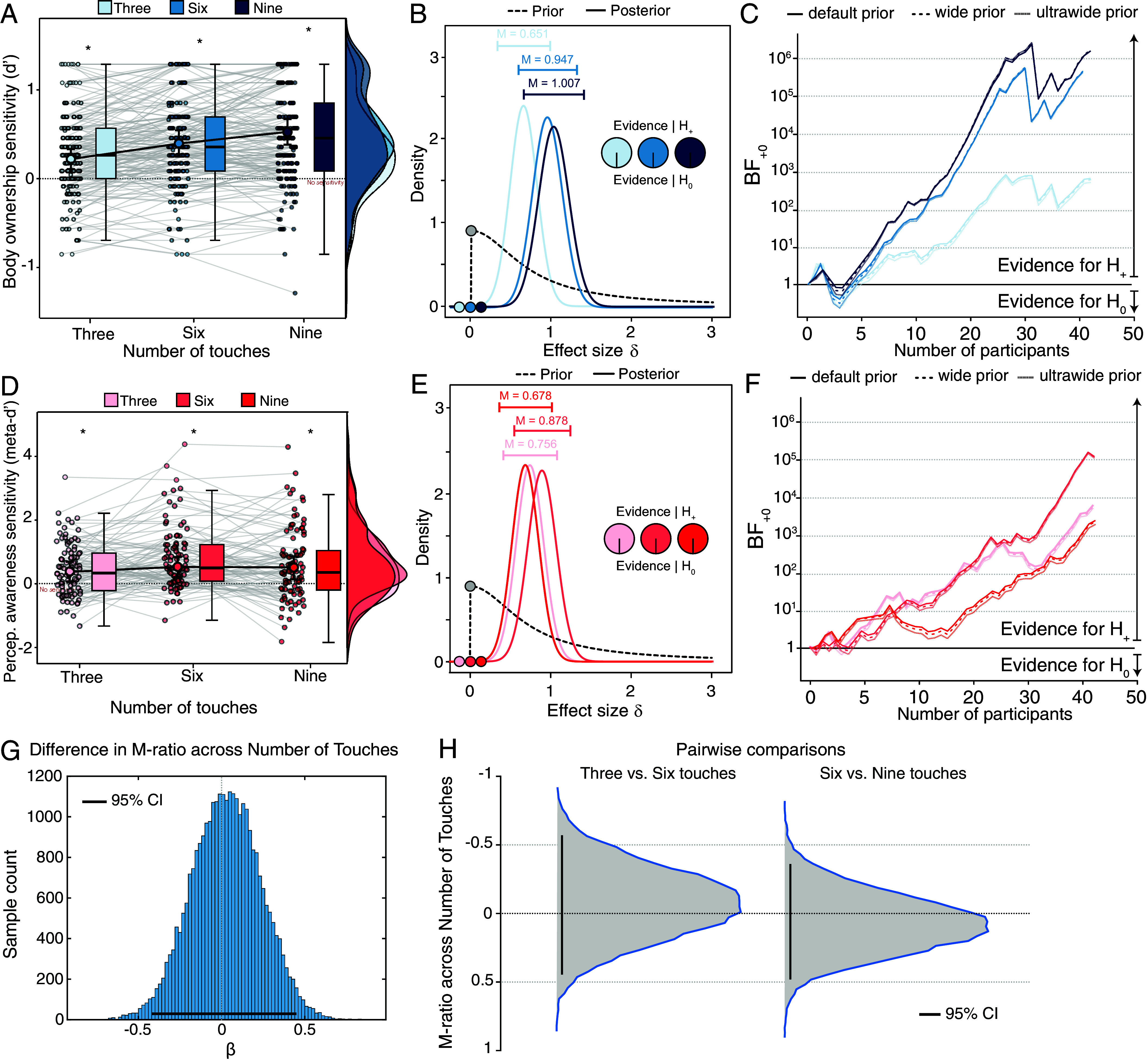
Psychophysical measures of body ownership and perceptual awareness sensitivity. (*A*–*C*) Body ownership sensitivity (d′). (*A*) Body ownership sensitivity increased with greater number of touches. Individual one-sample *t* tests against zero. Body ownership d′ was significantly above zero with three, six, and nine touches. Bayesian one-sample *t* tests against zero (one-tailed). (*B*) Prior and posterior distributions. Bayes factors offered overwhelming evidence in favor of the alternative hypothesis model with increasing numbers of touches (i.e., body ownership d′ > 0). The estimated population effect sizes, medians, and 95% central credible intervals for each number of touch condition are depicted. (*C*) Sequential analysis with robustness assessment. The evidence for the alternative hypothesis model is extremely stable across different prior distributions for each number of touches condition, ranging from 0.327 to 0.98 (r = 0.624) for three touches, from 0.588 to 1.313 (r = 0.94) for six touches, and from 0.64 to 1.38 (r = 1.004) for nine touches. (*D*–*F*) Perceptual awareness sensitivity (meta-d′) calculated using the maximum likelihood estimation method. (*D*) Perceptual awareness sensitivity was higher for six touches compared to three touches. Individual one-sample *t* tests against zero. Perceptual awareness meta-d′ was significantly above zero with three, six, and nine touches. Bayesian one-sample *t* tests against zero (one-tailed). (*E*) Prior and posterior distributions. Bayes factors offered overwhelming evidence in favor of the alternative hypothesis model with increasing numbers of touches (i.e., perceptual awareness meta-d′ > 0). Estimated population effect sizes, medians, and 95% central credible intervals for each number of touch condition are depicted. (*F*) Sequential analysis with robustness assessment. The evidence for the alternative hypothesis model is extremely stable across different prior distributions for each number of touches condition, ranging from 0.42 to 1.098 (r = 0.741) for three touches, from 0.528 to 1.235 (r = 0.878) for six touches, and from 0.351 to 1.011 (r = 0.654) for nine touches. (*G*) Estimation of differences in M-ratio between different numbers of touches, assessed with 95% HDI, suggests no difference between the numbers of touches. Each histogram represents posterior densities of M-ratio for all conditions. The results show a lack of difference (in log units) between condition posteriors. (*H*) Pairwise comparison of posterior distributions between numbers of touches conditions provided no evidence for differences in perceptual awareness efficiency across numbers of touches. Histograms represent posterior densities of M-ratio for all conditions. Error bars represent 95% CrI. Asterisks indicate significant differences from zero (*P < 0.05). Supplementary results replicating the effects of visuotactile asynchrony reported in Experiment 1 can be found in *SI Appendix*, Fig. S4.

Next, we investigated whether the relationship between body ownership and perceptual awareness observed in Experiment 1 would also hold during the gradual build-up of the RHI over repeated visuotactile stimulations. In Experiment 2, we used the same SDT metrics as in Experiment 1, but we varied the number of touches (3, 6, or 9) used to induce the RHI, thereby manipulating the amount of multisensory evidence that was accumulated. As expected, using a repeated-measures ANOVA, we found that body ownership sensitivity significantly increased with the number of touches $\begin{array}{c} \left(F(1.615,\;69.442)=22.323,P<0.001,\;\eta p^{2}=0.342\right) \end{array}$, showing that multisensory evidence accumulation during the build-up of the RHI enhances the sensitivity of body ownership to visuotactile asynchrony signals. We also replicated the Experiment 1’s finding that body ownership sensitivity significantly increased across degrees of visuotactile asynchrony (*F* (1.955, 84.044) = 43.179, *P* < 0.001, *ηp* ^2^ = 0.501) (Fig. 4 *A*–*C*). There was no interaction between the number of touches and the degree of visuotactile asynchrony $\begin{array}{c} (F(6,\;258)=1.631,P=0.139,\;\eta p^{2}=0.037), \end{array}$ consistent with the idea that sensory evidence accumulation relies on mechanisms beyond visuotactile integration. Notably, perceptual awareness sensitivity also significantly increases with the number of touches $\begin{array}{c} \left(F(2,\;86)=4.02,P=0.018,\;\eta p^{2}=0.089\right) \end{array}$ (Fig. 4 *D*–*F*), suggesting that multisensory evidence accumulation enhances the sensitivity of perceptual awareness to body ownership signal processing. We also found that perceptual awareness sensitivity significantly increased across degrees of visuotactile asynchrony $\begin{array}{c} \left(F(2.22,\;86.95)=22,P<0.001,\;\eta p^{2}=0.339\right) \end{array}$, replicating the findings of Experiment 1, and we did not find an interaction between number of touches and degree of visuotactile asynchrony $\begin{array}{c} \left(F(6,\;258)=1.027,P=0.408,\;\eta p^{2}=0.023\right) \end{array}$. In summary, the close relationship between objective body ownership discrimination and perceptual awareness reports is also observed as the RHI gradually grows stronger over varying numbers of visuotactile stimuli.

### The Proportion of Body Ownership Information Accessed by Perceptual Awareness Is Constant Across Varying Levels of Evidence Accumulation.

We calculated perceptual awareness efficiency (M-ratio) across different numbers of touches (Fig. 4 *G* and *H*) and degrees of visuotactile asynchrony (*SI Appendix*, Fig. S4 *L* and *M*). The posterior distribution of M-ratio did not vary across touch number conditions (Fig. 4*G*), nor were there significant differences between pairs of touch conditions (Fig. 4*H*), as all 95% HDIs encompassed zero. A Bayesian one-way ANOVA on the single-subject M-ratio indices supported the observed null difference across touch number conditions (*BF*_01_ = 5.87). This finding indicates that the amount of body ownership information available to perceptual awareness remains consistent across varying numbers of visuotactile stimuli, i.e., across different levels of sensory evidence accumulation. The posterior distribution of M-ratio did not vary across degrees of visuotactile asynchrony (*SI Appendix*, Fig. S4*L*), and no differences were observed between pairs of asynchrony conditions (*SI Appendix*, Fig. S4*M*). Together, these findings support the conclusion that the amount of multisensory information accessible by awareness is proportional to the available multisensory evidence and that this relationship remains stable as the RHI gradually emerges.

### Multisensory Evidence Accumulates for Body Ownership and Perceptual Awareness at Equivalent Rates.

In Experiment 2, we varied the amount of multisensory evidence by manipulating the number of touches (3, 6, or 9) while employing SDT metrics. However, SDT does not account for the temporal dynamics of perceptual decision-making, and the results are limited to the three predetermined numbers of touches investigated. DDM of perceptual decision response times and awareness report response times overcomes this limitation by allowing participants to “control” how much sensory evidence accumulates in each trial for the RHI to emerge. Thus, we designed Experiment 3 as a sped-up version of Experiment 2, in which participants received up to 30 s of visuotactile stimulation (up to 15 touches) and were instructed to indicate which rubber hand felt more like their own (body ownership discrimination) as soon as they detected a difference in hand ownership. PAS reports were collected immediately after this Type 1 report, which was also a sped-up task.

**Fig. 5.**
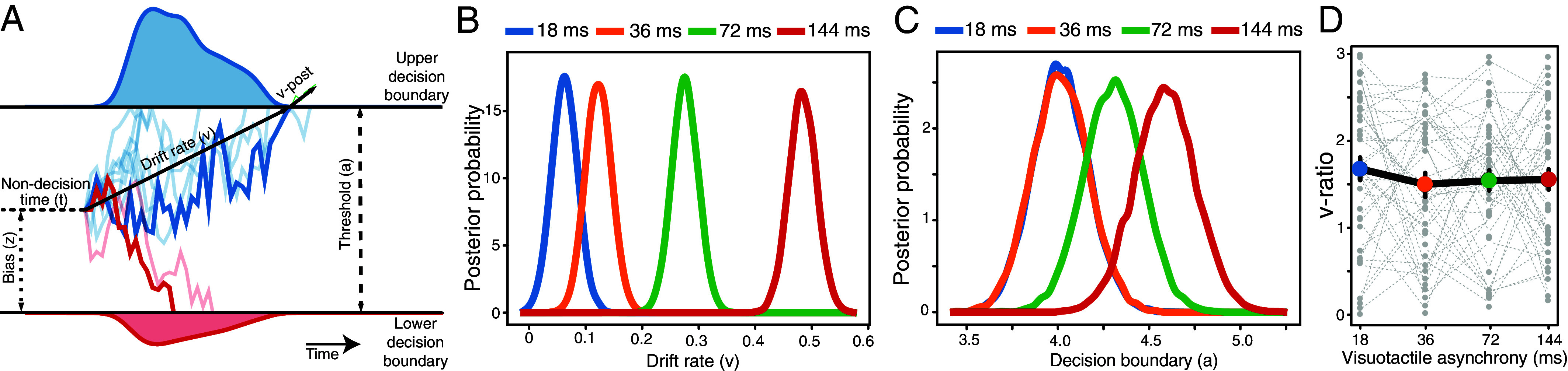
Hierarchical DDM and v-ratio measures of perceptual evidence accumulation. (*A*) DDM framework: the DDM predicts that sensory information accumulates over time until it reaches a decision boundary. Drift rate reflects the accumulation speed of perceptual evidence, bias represents the preference for a particular choice, nondecision time refers to processes unrelated to perceptual decision-making, and threshold represents the distance between decision boundaries, which determines the amount of evidence required to make a decision. (*B* and *C*) Hierarchical DDM posterior distributions: (*B*) Drift rate posterior distributions increased with higher visuotactile asynchrony, indicating that body ownership evidence was accumulated more rapidly in favor of the synchronously touched rubber hand as asynchrony increased. (*C*) Decision boundary posterior distributions increased with greater visuotactile asynchrony, suggesting that more perceptual evidence was required before committing to a response as the degree of asynchrony increased. (*D*) Mean v-ratio: this index of perceptual awareness efficiency showed no significant difference across visuotactile asynchrony levels, implying that conscious access to body ownership information remained stable. Error bars represent the standard error of the mean.

Using DDM parameters (Fig. 5*A*), we found that the rate of multisensory evidence accumulation (drift rate based on Type 1 response time) increased with greater degrees of visuotactile asynchrony difference between the two rubber hands (Fig. 5*B*), indicating more rapid accumulation of body ownership evidence over time when the difference in visuotactile asynchrony between the rubber hands was more pronounced. Additionally, we found a slight increase in the amount of multisensory evidence required to make a discrimination decision (decision boundary) with higher asynchronies (Fig. 5*C*), suggesting that participants may have taken slightly longer to respond, potentially explaining the overlapping response-time distributions (*SI Appendix*, Fig. S5 *A* and *B*).

Critically, we calculated the DDM-derived index *v-ratio* to investigate the relationship between the drift rate for body ownership discrimination and the drift rate for perceptual awareness. Similar drift rates would indicate a close relationship between body ownership and awareness. The v-ratio estimates perceptual awareness efficiency (conceptually equivalent to M-ratio, see above) while accounting for the dynamics of perceptual evidence accumulation (54). Importantly, no significant differences in v-ratio were found across levels of visuotactile asynchrony (Fig. 5*D*), indicating that the evidence accumulation processes for body ownership and perceptual awareness are comparable; that is, the proportion of accumulated body ownership information accessible to awareness is consistent with the accumulated multisensory evidence for body ownership. The findings from Experiment 3 are important because they provide complementary evidence supporting the close relationship between objective discrimination and perceptual awareness when directly analyzing the evidence accumulation process underlying perceptual decision-making in the current RHI paradigm.

## Discussion

The current study investigated bodily self-perception by distinguishing conscious and unconscious processes using psychophysics and computational modeling. Unexpectedly, and unlike what is known about the relationship between conscious and unconscious processes in vision and audition, we found a close relationship between perceptual awareness and objective perceptual discrimination of body ownership. Specifically, our findings show that the sensitivity of body ownership to visuotactile information (body ownership sensitivity) aligns with the sensitivity of perceptual awareness to body ownership processing (perceptual awareness sensitivity). Additionally, the proportion of body ownership information reaching consciousness (perceptual awareness efficiency) remained constant regardless of the amount of multisensory information integrated or accumulated. This finding reveals that perceptual awareness has constant and continuous access to body ownership information, reinforcing the notion of a consistent relationship. A control experiment using a visuotactile simultaneity task showed that strong conscious access is specific to body ownership and does not reflect multisensory integration in general. These results are conceptually important, as they suggest that the multisensory information the brain uses to generate bodily self-perception is inherently tied to conscious awareness, emerging directly in conscious awareness as bodily sensory signals are combined into a coherent multisensory own-body representation. This research advances our understanding of the relationship between conscious awareness and the sense of self with implications for consciousness research, body representation research, and applied neuroscience research into disorders of bodily awareness.

The current findings have significant theoretical implications for consciousness research, suggesting a fundamental distinction between body ownership information and unisensory visual (14, 16, 58–62), auditory (63, 64), or visuotactile (Control Experiment 2) information in their access to awareness, with important consequences for our understanding of bodily self-consciousness and how it diverges from our conscious processing of external sensory information. Unlike visual and auditory stimuli, which undergo some level of processing before reaching awareness (19, 20), and unlike certain multisensory interactions where unconscious signals in one sensory modality influence conscious perception in another modality (43, 44, 65, 66), our results suggest that body ownership perception does not follow the same principle. Across three experiments in which we varied the strength of the sense of body ownership in the RHI by manipulating the degree of multisensory integration (visuotactile asynchrony; Experiment 1) or the amount of sensory information accumulated during the emergence and gradual strengthening of the illusion (Experiments 2 and 3), we consistently observed a close coupling of objective body ownership discrimination with subjective perceptual awareness reports. Additionally, we consistently observed a constant relationship between the two, i.e., constant perceptual awareness efficiency, which indicates that conscious awareness has continuous access to body ownership information. Furthermore, since we found no reliable evidence for unconscious processing of body ownership, and conscious access was stronger in the body ownership task compared to a visuotactile integration control task (where unconscious effects were observed), this suggests that body ownership may enjoy a distinctively privileged status in awareness.

We see two possible explanations. One is the self-related nature of body ownership. Body ownership differs fundamentally from typical unisensory stimuli or multisensory stimuli that signal external events: It is egocentrically anchored in space, intrinsically self-related, and continuously present in conscious experience. Accordingly, body ownership may have privileged conscious access as this information contributes to generating a unified, continuous first-person representation of bodily self. The other plausible explanation relates to the complexity of the multisensory integration that underpins body ownership. Body ownership involves not only visuotactile integration in the temporal domain (as in our control task) but also visuoproprioceptive integration in the spatial domain, as well as inputs from other sensory modalities [e.g., thermosensory and nociceptive signals from the real hand remapped onto the rubber hand (67, 68)]. These diverse signals are combined into a rich, multifaceted, multisensory experience of one’s own limb. While both body ownership and visuotactile simultaneity judgments rely on Bayesian causal inference (2, 6, 34, 37, 69–71), body ownership requires integration across multiple sensory dimensions and spatiotemporal scales, arguably making it more complex than the visuotactile integration in simple simultaneity judgments. This more complex integration process may require substantial information integration across widespread brain regions and the allocation of significant cognitive resources, requiring conscious awareness. This view aligns with the neuroimaging studies that have associated changes in the sense of body ownership during RHI paradigms with activations in distributed and functionally interconnected premotor, posterior parietal, lateral occipital, and insular regions (5, 37, 72–74). According to GNWT, these activations indicate conscious awareness due to the activation of widespread frontoparietal networks (75, 76). Thus, we propose that the complex processing of integrated multisensory information in body ownership has already surpassed the threshold for “ignition” and “broadcasting” according to GNWT, meaning that multisensory body ownership perception is intrinsically tied to conscious awareness. This interpretation is supported by the consistent level of perceptual awareness efficiency [a measure of conscious access (77–80)] across different degrees of visuotactile stimulation asynchrony and varying levels of multisensory evidence accumulation, suggesting that the proportion of body ownership information accessed by awareness is stable, regardless of the quantity of visuotactile information processed.

Our findings regarding conscious access to body ownership information have significant implications for our understanding of selfhood and bodily self-awareness. It provides an empirical foundation for the view that our sense of body ownership is a constant presence in conscious awareness, grounding our perception and experiences in a consistent physical self, or, as William James put it, “the feeling of the same old body always there” (1). The current findings also provide empirical support for theories of bodily self-consciousness (81, 82) that have posited a central role for multisensory integration and the bodily self in consciousness, although previous experimental evidence for this connection has been limited. In contrast to visual information about external objects and events, which strongly relies on stimulus characteristics and top–down amplification to enter awareness, we propose that information pertaining to body ownership is always afforded access to awareness owing to its fundamental self-related nature and the complexity of the underlying multisensory integration processes. This embedding of body ownership within conscious awareness may stem from the crucial role of maintaining a coherent and continuous conscious sense of self for survival, surpassing the importance of continuous conscious access to external stimuli.

Our study revealed that body ownership and perceptual awareness sensitivity are strongly influenced by brief visuotactile asynchronies, with asynchronies as short as 30 ms significantly affecting participants’ awareness reports during the RHI. This finding enhances our understanding of how visual and tactile signals integrate to create the experience of body ownership (83, 84), suggesting that body ownership sensitivity and perceptual awareness sensitivity emerge together. It also establishes minimal visuotactile asynchrony thresholds, indicating how finely the system is tuned to detect body ownership information; the more attuned the system is, the shorter the asynchronies that can produce above-chance sensitivity. These insights offer potential for studying neuropsychiatric disorders such as schizophrenia (85–88), eating disorders (89, 90), borderline personality disorder (91, 92), and autism-spectrum disorders (93–95), where a distorted sense of body ownership is observed. Future research could explore variations in these minimal asynchrony thresholds to determine whether impairments stem from disrupted multisensory integration or from how these signals enter conscious awareness.

We found no conclusive evidence of unconscious processing in our body ownership experiments, e.g., deviations of type-1 and type-2 sensitivity for difficult-to-discriminate stimuli (i.e., trials with narrow visuotactile asynchronies) or changes in M-ratio. Although the raw M-ratios were lower than 1 (typically in the range of 0.6 to 0.8), indicating suboptimal perceptual awareness efficiency (77), this alone does not constitute evidence for unconscious processing, as reduced efficiency may also result from variability in subjective report criteria, additional noise in perceptual awareness judgments, metacognitive biases, or performance-related factors. This raises the possibility that body ownership may be fully implemented at the level of conscious processing. However, this interpretation requires further testing. For example, future studies could examine the relationship between body ownership processing and perceptual awareness in the 18 to 31 ms range, which was not investigated here, using more advanced robotic setups capable of applying visuotactile stimulation asynchronies with 1-ms precision. In addition, future studies should also systematically manipulate the degree of spatial congruence between the visual and tactile stimuli to assess whether the present findings hold when spatial information related to body ownership is varied. It would also be valuable to explore whether the current findings generalize to full-body illusion paradigms, in which the sense of owning one’s entire body rather than a single limb is manipulated (96–98).

Our findings also hold significant implications for body representation research. First, they link multisensory integration to the conscious perception of illusory body ownership, providing empirical support for a connection central to current theories of body ownership and the bodily self (5, 6, 34, 82, 99) but not previously tested directly. Second, they strongly suggest that the changes in sensitivity and psychometric functions observed in earlier RHI psychophysical paradigms are closely tied to the subjective experience of body ownership rather than being driven by unconscious guessing at above-chance levels (2, 35–37). Third, our findings provide insight into how the RHI emerges and strengthens with repeated touches, demonstrating how the illusion develops through a gradual accumulation of sensory evidence—an important yet understudied aspect of bodily illusion (55, 56). Fourth, our study introduces a methodological approach that offers significant advantages over conventional approaches relying on questionnaires and indirect behavioral measures (e.g., “proprioceptive drift”) in body ownership research. SDT analysis and computational modeling of perceptual awareness (metacognition) and sensory evidence accumulation enable objective and independent assessments of multisensory signal processing, conscious access, and estimates of perceptual (100–103) and metacognitive biases (51, 77, 101), facilitating future research into dissecting the sense of body ownership into its constituent processes. Furthermore, this method can be adopted to study other bodily illusions (29, 96, 104–108), enabling researchers to explore conscious and unconscious processing in other aspects of body representation, such as the perception of body size, shape, and sense of limb movement.

In conclusion, our findings reveal a close relationship between conscious awareness and multisensory integration in body ownership. The sensitivity and rate of evidence accumulation for conscious awareness of body ownership mirror those for body ownership based on multisensory information. Furthermore, the proportion of body ownership information accessed by conscious awareness remains consistent regardless of the level of multisensory integration and the amount of sensory evidence accumulation, providing evidence for constant and continuous conscious access. Collectively, these findings underscore a fundamental connection between conscious awareness and body ownership, suggesting a unique role for the bodily self in consciousness and indicating that body ownership information processing is especially dependent on conscious awareness.

## Materials and Methods

### Experiment 1.

#### Participants.

Thirty-eight naïve participants were recruited; 32 (17 females; *M*_age_ = 28.13, *SD* = 5.1, range 20 to 40) who experienced the RHI (*Inclusion Test*) completed the experiment; inclusion criteria were based on previous studies (5, 35, 36, 49, 50, 109–112). Sample size was determined by a priori power analysis for a repeated-measures ANOVA with five asynchrony levels, targeting 0.8 power to detect a $\eta p^{2}$ = 0.4 at α = 0.05. This effect size is smaller than previously reported for body ownership sensitivity ($\eta p^{2}$ = 0.891 to 0.923). Power analyses were run in R (*pwrss*, v0.3.1).

All experiments were approved by the Swedish Ethical Review Authority (Dnr 2021-03164). All participants gave informed consent in accordance with the Declaration of Helsinki and received 150 SEK per hour for their participation.

#### Inclusion test.

Not all participants experience the RHI (5, 109) due to individual differences in multisensory processing (29). Each underwent a standardized assessment (30). A lifelike prosthetic right hand (Fillauer® Model 30916-R, plaster-filled) was placed 15 cm above the hidden real hand and stroked synchronously for 12 s at 0.5 Hz. Inclusion required a) a mean score > 1 on ownership items (Q1 to Q3) and b) a difference > 1 between ownership and control items (Q4 to Q9; see *SI Appendix*, Table S1). Only those meeting criteria continued to the main experiment.

#### Stimulation and apparatus.

Participants rested the right-hand palm-down beneath a wooden platform (30 cm from midline). Two identical prosthetic right hands (Fillauer® Model 30916-R) were placed 5 cm apart, angled 30°, with a fixation point between them (35, 36, 113). The right arm rested on an Ergorest^®^ support; head position was stabilized by a chin rest. Three robotic arms (custom-built) applied tactile stimuli to real and rubber hands using 7 mm probes (35, 36, 49, 50). Movement was driven by HS-7950TH UltraTorque servos, monitored via E3X-HD41 fiber sensors (OMRON^®^) for precise tap timing. The applied and theoretical degrees of asynchrony were confirmed to be similar through laser verification. White noise delivered via earphones masked motor sounds.

#### Psychophysical task.

Participants fixated centrally while six taps were applied to each index finger within 12 s, randomly distributed over five finger sites. One rubber hand was tapped synchronously and the other asynchronously (18, 31, 52, 88, 150 ms). After an auditory cue, participants completed a 2AFC task indicating which hand felt most like their own (3 s window), then rated the clarity of ownership using a 3-point Perceptual Awareness Scale (PAS: “unclear,” “vague,” “clear”) (79). Between trials, they moved their fingers to break the illusion. Each participant completed 300 trials (six blocks of 60).

#### Body ownership sensitivity analysis.

Type-1 SDT quantified sensitivity (d′) and bias to body ownership signals. Hits were defined as reporting the right hand as “own” when it was synchronously tapped, and false alarms (FAs) as reporting the right hand as “own” when asynchronously tapped. The d′ value was calculated using the formula $d_{\text{ownership}}^{\prime }=\left(\frac{1}{\sqrt{2}}\right)\left(Z\left(P_{\mathrm{Hit}}\right)-Z\left(P_{\mathrm{FA}}\right)\right)$. *A d′ of zero* indicates chance-level discrimination; higher values reflect stronger sensitivity. Bias was estimated as $C_{\mathrm{rubber}\;\mathrm{hand}}=-\left(\frac{1}{2}\right)\left(Z\left(P_{\mathrm{Hit}}\right)+Z\left(P_{\mathrm{FA}}\right)\right)$. Positive/negative values denote left/right bias, respectively.

#### Perceptual awareness sensitivity (meta-d′).

Using maximum-likelihood estimation (51, 114), we derived meta-d′ and metacognitive bias indices from PAS responses. *Meta-d′* of *0* indicates no relationship between awareness and performance; positive values indicate alignment between subjective and objective sensitivity.

#### Perceptual awareness efficiency (M-ratio).

Perceptual awareness efficiency was quantified via Bayesian hierarchical modeling (52) using JAGS with three 10,000-iteration MCMC chains (1,000 burn-in). *M-ratio* (0 to 1) represents awareness efficiency controlling for d′ and bias. Group-level posterior distributions were compared using 95% HDIs; intervals including zero indicated no difference.

#### Statistical analyses.

Analyses were performed in MATLAB R2023b using customized code. Frequentist and Bayesian repeated-measures ANOVAs assessed *d′*, *meta-d′*, and *M-ratio*, with Greenhouse–Geisser correction where required. Bayes factors used uniform priors (*r* = 0.5 fixed, 1 random, 0.354 covariates). Bayesian *t* tests employed Cauchy priors centered at 0 (width = 0.707). Sensitivity analyses tested model robustness.

### Control Experiment 1.

#### Participants.

Thirty-five were recruited; 32 (16 females; *M*_age_ = 26.25, *SD* = 7.81, range 19 to 39) who experienced a vivid RHI participated.

#### Stimulation and task.

Identical apparatus and procedures were used, except the rubber hands were rotated 90° clockwise, violating the spatial rule of the RHI and abolishing ownership (5, 31, 57). Participants performed the same psychophysical task.

#### Analyses.

Body ownership sensitivity (*d′*) was analyzed as in Experiment 1.

### Control Experiment 2.

#### Participants.

Thirty-three were recruited; 32 (18 females; *M*_age_ = 27.52, *SD* = 4.86, range 20 to 40) provided valid type-2 SDT data.

#### Stimulation and task.

Procedures matched Experiment 1 except that two wooden blocks (30 × 9.7 × 6.5 cm) replaced the rubber hands. Participants reported which block was tapped synchronously and rated clarity via PAS. This setup allowed assessment of visuotactile simultaneity perception without body ownership (32, 55).

#### Analyses.

Visuotactile simultaneity sensitivity was computed from simultaneity judgments.

### Experiment 2.

#### Participants.

Fifty-three were recruited; 44 (25 females; *M*_age_ = 29.64, *SD* = 6.2) passed inclusion. Power analysis (four asynchrony × three touch levels) targeted 0.8 power for partial $\eta p^{2}$ = 0.4 at α = 0.05 (*pwrss*, v0.3.1).

#### Stimulation and task.

Identical to Experiment 1 except stimulation duration varied (6, 12, 18 s equivalent to 3, 6, 9 touches, respectively). The psychophysical procedure was otherwise unchanged.

#### Analyses.

Conducted as in Experiment 1 with the addition of “number of touches” as a within-subject factor.

### Experiment 3.

#### Participants.

Ninety-nine were recruited; 75 met inclusion, and after excluding those with >5% missing trials, 65 (44 females; *M*_age_ = 27.98, *SD* = 6.37) remained for analysis. Due to technical issues with the custom software for collecting postdecisional response times, only 41 participants contributed to the v-ratio dataset. Both samples exceeded the required *N* = 40 threshold for power.

#### Psychophysical task.

Procedures mirrored Experiment 1 except that stimulation lasted for up to 30 s (≈15 touches). Participants responded as soon as they experienced ownership; stimulation ceased immediately. They then gave PAS ratings within 3 s. Each completed 480 trials (eight blocks × 60).

#### Response time analyses.

Decision response time (from first touch to 2AFC response) and postdecision time (from 2AFC response to PAS rating) were computed per condition. Non-normal data (Kolmogorov–Smirnov test) were analyzed using nonparametric alternatives (Kruskal–Wallis).

#### Drift diffusion modeling.

Body ownership decision processes (115, 116) were decomposed using hierarchical DDM [HDDM; (117)] implemented in Docker (118). Parameters estimated: starting point (*z*), nondecision time (*t*), drift rate (*v*), and decision boundary (*a*). MCMC sampling (20,000 samples, 1,000 burn-in) estimated posterior distributions. Convergence was confirmed via trace inspection, autocorrelations, and Gelman–Rubin *Rˆ* < 1.02.

#### Perceptual awareness evidence accumulation.

Evidence accumulation for conscious awareness was quantified by *v-ratio* (54), i.e., postdecisional drift rate (PAS)/drift rate (2AFC), representing dynamic perceptual awareness efficiency integrating multisensory evidence accumulation.

#### Statistical analyses.

Conducted in R (v4.3) (119) and JASP (v0.18) (120). ANOVAs examined effects of asynchrony, number of touches, and DDM parameters, followed by Tukey-HSD post hoc tests.

Additional methodological details are provided in *SI Appendix*.

## Supplementary Material

Appendix 01 (PDF)

## Data Availability

The data along with custom code can be accessed through the Open Science Framework (https://osf.io/fm96c/) (121).
