# Supplementary material for: Conscious awareness, sensory integration, and evidence accumulation in bodily self-perception
Source: Proc Natl Acad Sci U S A. 2025 Dec 3;122(49):e2503629122. doi: 10.1073/pnas.2503629122 (PMC12704745; doi:10.1073/pnas.2503629122)
Supplement: Supplementary file 1 — Appendix 01 (PDF) [file pnas.2503629122.sapp.pdf]

SUPPLEMENTARY INFORMATION

Conscious awareness, sensory integration, and evidence accumulation in bodily self-perception

Renzo C. Lanfranco, Sucharit Katyal, August Hägerdal, Xiaole Luan, Victoria Nos, & H. Henrik Ehrsson

Corresponding authors: Renzo.Lanfranco@ki.se and Henrik.Ehrsson@ki.se

Supplementary Information: Materials and Methods

Experiment 1

Participants. We initially recruited 38 naïve participants. In line with our a priori inclusion criterion based on previous studies (1–9), 32 participants (17 females; M_{age} = 28.13, SD_{age} = 5.1; age range, 20–40) who could experience the RHI (see Inclusion test below) took part in the main experiment. The sample size was supported by an a priori power analysis for a repeated-measures analysis of variance (ANOVA), with five degrees of visuotactile asynchrony conditions aiming at obtaining 0.8 statistical power to detect an effect size of 0.4 partial eta-squared at a standard 0.05 alpha error probability. Importantly, this effect size is far smaller than the effect sizes we have previously reported for body ownership sensitivity (d'), with partial eta-squared values of 0.891 (4) and 0.923 (5) when very similar experimental designs were used for visuotactile asynchrony manipulation. Because no prior studies have assessed perceptual awareness/metacognitive sensitivity in a psychophysical task of body ownership, we employed larger sample sizes. This power analysis was run using the pwrss R package, version 0.3.1.

All experiments were approved by the Swedish Ethical Review Authority (Dnr 2021-03164). All participants gave informed consent in accordance with the Declaration of Helsinki and received 150 SEK per hour for their participation.

Inclusion test. Not all participants are susceptible to the RHI (3, 6), presumably due to individual differences in multisensory processing and perceptual priors (10). These illusion non-responders cannot perform the current psychophysical tasks or produce responses that can be fitted with psychometric functions or models (2). Therefore, before being included in the main experiments, participants underwent assessment to determine their ability to experience the RHI through a standardised procedure. Each participant was seated before a table, and their right hand was positioned on a support surface. A small table, situated 15 cm above their hand, presented a lifelike prosthetic right hand (Model 30916-R, Fillauer®, filled with plaster) positioned congruently with their hidden real hand. With their gaze fixed upon the rubber hand, participants experienced synchronised stroking from the experimenter, who delicately applied strokes to both the rubber hand and the real hand for a duration of 12 seconds, aligning the timing of each stroke as closely as possible. Each stroke lasted for 1 s and targeted the same locations on the index fingers as in Experiment 1, maintaining a frequency of 0.5 Hz. The participants subsequently completed the RHI questionnaire utilised by Botvinick and Cohen (11). Inclusion criteria were established, requiring (a) a mean score on ownership statements (Q1, Q2, and Q3) exceeding 1,

and (b) a discrepancy of more than one between this mean score and the mean score of control items (Q4–Q9; see **Table S1**). Participants meeting these predetermined criteria were then invited to participate in the respective experiment.

Stimulation and apparatus. The participants were instructed to place their right hand, palm facing down, on a flat surface beneath a wooden platform, approximately 30 cm from their body's midline. Two identical right cosmetic prosthetic hands (Model 30916-R, Fillauer®, filled with plaster), were positioned on the platform above the participant's real hand, which was hidden from view. The prosthetic hands (referred to as "rubber hands") were angled upwards by 30 degrees at the front. Positioned side by side and equidistant (5 cm) from the participant's real hand, the rubber hands allowed simultaneous and independent induction of the RHI on each of the two fake hands (2, 4, 12). A white circular fixation mark was placed precisely between the rubber hands to assist participants in maintaining focus. To minimise head movement, the participants used a chin rest, whereas an elbow rest (Ergorest Oy®, Finland) supported their right arm, ensuring comfort and relaxation throughout the experiment.

Tactile stimuli were administered to both the rubber and real hands via three robotic arms (1, 2, 4, 5). Each arm comprised two 17-cm-long and 3-cm-wide metal pieces, along with a metal slab measuring 10 × 20 cm. Joint movement was facilitated by two HS-7950TH UltraTorque servos, while another servo powered the proximal and support components. A 7-mm diameter touch probe was affixed to the end of the distal metal piece, enabling precise contact with the hands during stimulation. The timing of taps was accurately recorded by E3X-HD41 fibre sensors (OMRON®, Netherlands), which measure the duration of the rebound of the laser light upon contact with the touch probes. The applied and theoretical degrees of asynchrony were confirmed to be similar through laser verification. To minimise external distractions and mask the sounds of the robotic motors, the participants wore earphones playing individually adjusted levels of white noise.

Psychophysical task. The participants fixated on a central marker while a robotic arm tapped their index fingers six times within a 12-s interval. To prevent skin irritation, the taps were randomised across five locations on the index finger: proximal to the nail on the distal phalanx, on the distal interphalangeal joint, on the middle phalanx, on the proximal interphalangeal joint, or on the proximal phalanx. Two robotic arms simultaneously tapped the corresponding locations on the two rubber hands: one synchronously with the touches on the real hand and the other at one of five asynchrony levels (18, 31, 52, 88 and 150 ms). In half of the trials, the left rubber hand was subjected to this asynchrony, whereas the right rubber hand was subjected to it in the remaining half. Following an auditory cue, the participants performed a two-alternative forced-choice (2AFC) task, indicating within a 3-second window which rubber hand (left or right) felt most like their own. They then evaluated the clarity of their sense of ownership towards the chosen rubber hand using a 3-point Perceptual Awareness Scale (13), selecting between "unclear experience," "vague experience," or "clear experience" within another 3-s period. Afterwards, the participants wiggled their fingers several times to break the illusion and reduce carry-over effects and then relaxed their fingers for the remainder of the 4 s rest period before the next trial began. A total of 300 trials were conducted, with an even distribution across the visuotactile asynchrony conditions, randomised across six blocks of 60 trials. The participants were offered a voluntary break after each block.

Body ownership sensitivity analysis. We employed Type-1 SDT analysis to examine changes in the RHI's sensitivity to body ownership signals, specifically the information conveyed by visuotactile asynchronies and bias across different levels of visuotactile asynchrony. We first

analysed the data from each participant individually, obtaining sensitivity and bias indices at the first level. We then performed group analysis at the second level on the basis of these indices.

To assess sensitivity to body ownership signals, we used a bias-independent index denoted as body ownership sensitivity or d'. Hits were categorised as trials where participants reported the right rubber hand as feeling most like their own when the right rubber hand was synchronously tapped with the real hand. False alarms (FAs) were defined as trials in which participants reported the right rubber hand as feeling most like their own when the left rubber hand was synchronously tapped with the real hand. The d' value was calculated using the 2AFC formula: $d'_{ownership} = \left(\frac{1}{\sqrt{2}}\right)(Z(P_{Hit}) - Z(P_{FA}))$, with $Z(P_{Hit})$ and $Z(P_{FA})$ representing the Z-scores associated with the probabilities of hits and FAs, respectively (14, 15). To prevent zero counts, we applied padding (edge correction) by adding or subtracting half a trial (14). A d′ value of zero indicates that body ownership could not distinguish between the synchronously tapped and asynchronously tapped rubber hand. Values greater than zero indicate above-chance discrimination, with higher values reflecting greater sensitivity to visuotactile asynchrony (see **Table S2** for by-condition means and standard deviations). Bias, reflecting a participant's general tendency to report illusory ownership over the left or right rubber hand, was estimated using the following formula for decision criterion: $C_{rubber\ hand} = -\left(\frac{1}{2}\right)(Z(P_{Hit}) + Z(P_{FA}))$. Positive and negative values denote bias towards the left and right rubber hands, respectively.

Perceptual awareness sensitivity analysis. To determine how sensitive each participant’s perceptual awareness judgement (PAS rating; see **Fig. S1C**) was to their body ownership sensitivity performance (perceptual awareness sensitivity, also referred to as metacognitive sensitivity or meta-d’) and the bias in such judgements (perceptual awareness bias, also referred to as metacognitive bias), we employed the maximum likelihood estimation procedure (16, 17). A meta-d′ value of zero indicates that subjective reports are unrelated to body ownership discrimination performance, reflecting no perceptual awareness sensitivity. Values above zero indicate that subjective reports align with discrimination performance, with higher values reflecting greater perceptual awareness sensitivity.

Perceptual awareness efficiency analysis. To assess the level of conscious access that each participant had across visuotactile asynchrony conditions, while controlling for task performance, we quantified perceptual awareness efficiency (also referred to as metacognitive efficiency or M-ratio) using a Bayesian hierarchical framework (18) that employs Markov chain Monte Carlo (MCMC) sampling to compute posterior distributions. We used three chains of 10,000 iterations with 1000 burn-in samples, with default parameters from JAGS, ensuring that convergence of the model’s MCMC chains was achieved (see **Fig. S1D**). Theoretically, M-ratio adopts values from 0 to 1, indicating the extent to which perceptual awareness ratings discriminate between the feeling of ownership induced by synchronously touched and asynchronously touched rubber hands (19). M-ratio controls for differences in first-order performance (body ownership d’) and bias. To test whether M-ratio differed between degrees of visuotactile asynchrony, group-level M-ratio posterior distributions (in log units) were calculated; we assessed whether their 95% high-density intervals (HDIs) encompassed zero or not (20), wherein intervals that encompass zero are interpreted as evidence for the absence of a difference (18).

Statistical analyses. Data analyses were conducted via MATLAB (R2023b, The MathWorks, Inc.) with customised code, incorporating methods adapted from Maniscalco and Lau (16) (https://www.columbia.edu/~bsm2105/type2sdt/) and Fleming (18) (https://github.com/metacoglab/HMeta-d). Body ownership sensitivity and perceptual

awareness sensitivity were examined via both frequentist and Bayesian repeated-measures analysis of variance (ANOVA), alongside one-sided one-sample t-tests against a null hypothesis of zero. In instances where Mauchly's test indicated a violation of sphericity, adjustments to degrees of freedom were made using Greenhouse–Geisser correction. Bayes factors were computed to evaluate the strength of evidence supporting both the null and alternative hypothesis models. For this purpose, a uniform prior distribution was employed, with r-scale coefficients set to 0.5 for fixed effects, 1 for random effects, and 0.354 for covariates in Bayesian repeated-measures ANOVA models. Additionally, a Cauchy prior distribution, centred around zero with a width parameter of 0.707, was utilised for Bayesian t-tests. Sensitivity analyses of priors were conducted to assess the robustness of the Bayesian models' prior distributions.

Control experiment 1

Participants. We initially recruited 35 naïve participants, but 32 participants (16 females; M_{age} = 26.25, SD_{age} = 7.81; age range, 19–39) experienced a vivid RHI and therefore took part in the control experiment.

Stimulation, apparatus, and psychophysical task. The experimental setup and apparatus utilised remained consistent with those employed in the main experiment. However, a pivotal alteration was introduced in this control experiment, where the rubber hands underwent a rotation of 90 degrees clockwise. This manipulation violates the spatial rule of the RHI since both rubber hands are placed in anatomically implausible orientations. Prior research has established that this manipulation effectively abolishes the RHI (3, 21, 22).

Analyses. Body ownership sensitivity was analysed as in the main experiment. However, because the RHI was effectively abolished in the control experiment, the PAS data could not be fitted by computational models of perceptual awareness/metacognitive sensitivity or perceptual awareness/metacognitive efficiency.

Control experiment 2

Participants. We initially recruited 33 naïve participants, but 32 participants (18 females; M_{age} = 27.52, SD_{age} = 4.86; age range, 20–40) provided data that could be fit by type-2 SDT analysis.

Stimulation, apparatus, and psychophysical task. The experimental setup and apparatus utilised remained consistent with those employed in the main experiment. However, the rubber hands were replaced by two rectangular blocks of wood of similar dimensions (30 cm length, 9.7x cm width, and 6.5 cm height). Moreover, participants were tasked with reporting which block was tapped synchronously with their own hand in each trial (i.e., visuotactile simultaneity judgment) and the clarity of their visuotactile simultaneity experience using a PAS scale. Since the blocks of wood break RHI (23, 24), this experiment allowed us to assess visuotactile simultaneity perception and awareness in the absence of body ownership changes.

Analyses. Visuotactile simultaneity sensitivity was assessed based on participants' judgments of visuotactile simultaneity.

Experiment 2

Participants. We initially recruited 53 naïve participants, and following our inclusion criterion, 45 participants (25 females; M_{age} = 29.64, SD_{age} = 6.2) who could experience the RHI (see Inclusion test above) were included in Experiment 2. The sample size was determined by an a priori power analysis for a repeated-measures ANOVA, with four levels of visuotactile asynchrony and three levels of number of touches, aiming to achieve a statistical power of 0.8 to detect a partial eta-squared effect size of 0.4 at a standard alpha error probability of 0.05. Notably, this effect size is significantly smaller than the effect sizes we have previously observed for body ownership sensitivity, where partial eta-squared values of 0.891 (4) and 0.923 (5) were found using similar experimental designs for visuotactile asynchrony manipulation. Since no prior studies have examined the effect of the number of touches, perceptual awareness, or metacognitive sensitivity in a psychophysical task of body ownership, we opted for a larger sample size. The power analysis was conducted using the pwrss R package, version 0.3.1.

Stimulation and apparatus. The stimulation and apparatus were the same as those in Experiment 1, with the exception that tactile stimulation was applied for 6, 12, or 18 s, corresponding to 3, 6, or 9 touches, respectively.

Psychophysical task. Aside from varying the number of touches during visuotactile stimulation, the task was the same as that in Experiment 1.

Analyses. All these analyses were conducted as in Experiment 1, with the addition of the number of touches as a new factor.

Experiment 3

Participants. We initially recruited 99 naïve participants, 75 of whom met the predefined inclusion criteria for being able to experience the RHI (see Inclusion Test) and were included in Experiment 3. Data from participants who failed to provide responses in more than 5% of the trials were excluded, resulting in a final sample of 65 participants (44 females; M_{age} = 27.98, SD_{age} = 6.37) for analysis. Owing to technical issues with the custom software for collecting post-decisional response times, data from the first 24 participants could not be properly recorded, leaving 41 participants for the v-ratio analysis. Importantly, both samples exceeded the minimum threshold of 40 participants, ensuring sufficient statistical power. Notably, drift rate estimations remained consistent across HDDM and v-ratio implementations, irrespective of sample size differences (see **Fig. S5C-D**).

Psychophysical task. Experiment 3 had a very similar design to that of Experiment 1, with two modifications: First, the visuotactile stimulation in Experiment 3 lasted for up to 30 s (i.e., 15 touches), and the participants were instructed to respond the 2AFC task as soon as they experienced rubber hand ownership. Visuotactile stimulation ceased immediately after the participants made their selection in the sped-up task. Next, they were instructed to respond to the 3-point PAS as quickly as possible, with a 3-s window. A total of 480 trials were conducted, evenly distributed across the visuotactile asynchrony conditions and randomised over eight blocks of 60 trials, as in Experiment 2. The participants were given the option to take a break after each block.

Response time analysis. We calculated the mean decision response time for all degrees of asynchrony and the number of touches conditions. This refers to the interval between the onset of the first touch on the participant's hand and their response to the 2AFC task. Additionally, we computed the post-decision response time, defined as the duration between the participant's response to the 2AFC task and their subsequent PAS report. When the Kolmogorov–Smirnov test revealed a violation of normality, the appropriate nonparametric test was used. For example, the Kruskal–Wallis test was applied as the nonparametric alternative to one-way ANOVA (the parametric tests are provided in the Supplementary Information).

Body ownership drift rate analysis. To disentangle the perceptual decision-making processes assessed by our body ownership discrimination task, we utilised DDM, which describes how information is accumulated until an internal decision threshold is reached, leading to a perceptual judgement (25, 26). This approach allowed us to break down body ownership perception into four key components: the starting point (z), which reflects any pre-decision bias between the two choice boundaries; the nondecision time (t), accounting for processes unrelated to the decision itself; the drift rate (v), representing the rate at which information accumulates in favour of one choice boundary; and the decision boundaries (a), which signal the moment when enough evidence has been gathered to make a decision. We applied a Bayesian hierarchical variant of DDM (HDDM (27)), implemented via the Docker HDDM framework (28). To estimate the posterior distributions of the model parameters, we employed Markov chain Monte Carlo (MCMC) sampling, generating 20,000 samples and discarding 1,000 samples during the burn-in phase. We assessed model convergence through visual inspection of trace plots and autocorrelation functions, along with the computation of the Gelman–Rubin R-hat statistic, ensuring that R-hat values were under 1.02.

Perceptual awareness evidence accumulation analysis. To assess evidence accumulation for conscious awareness, we calculated v-ratio (29), which corresponds to the ratio of the drift rate for body ownership discrimination to the post-decisional drift rate – the PAS report. This v-ratio serves as a dynamic alternative to M-ratio, i.e., a measure of metacognitive or perceptual awareness efficiency that incorporates evidence accumulation processes.

Statistical analyses. Data analysis was performed using R (30) with custom scripts, and JASP (31). To examine differences in response time, drift rate, decision boundary, non-decision time, and v-ratio across varying degrees of visuotactile asynchrony, we performed ANOVA models, followed by post-hoc comparisons using Tukey's honest significant difference method.

Supplementary Information: Results

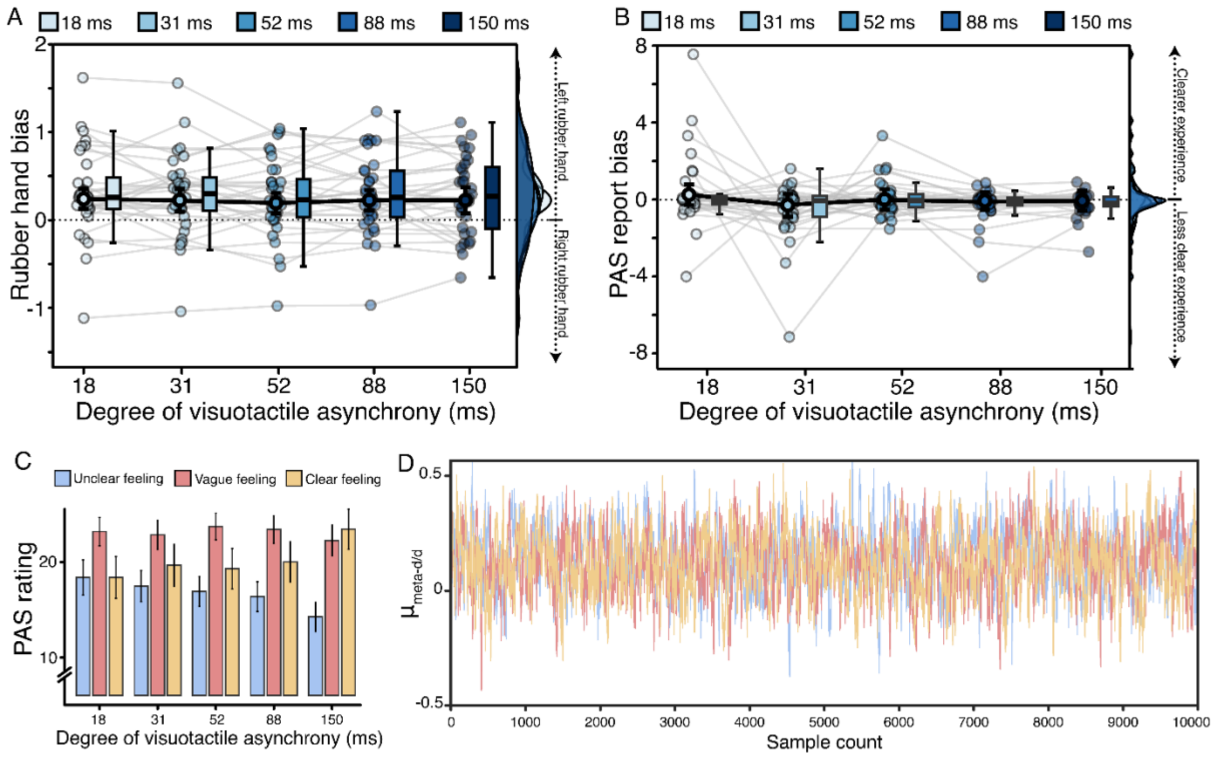

Figure S1. Supplementary Results of Experiment 1: (A) Rubber hand bias results. Rubber hand bias did not vary significantly across degrees of visuotactile asynchrony ($F_{(4,\,124)} = 0.375, p = .826$), but it was significantly above zero with all degrees of visuotactile asynchrony (all $p <$ 0.004), showing a consistent bias favouring the rubber hand closer to the body midline (left rubber hand). (B) PAS report bias results. Metacognitive bias did not vary significantly across degrees of visuotactile asynchrony ($F_{(2.28,\,70.53)} = 2.34, p = .091$). PAS report bias did not depart significantly from zero with any degrees of visuotactile asynchrony (all $p > 0.057$), which suggests that there were no consistent biases during the PAS ratings. (C) PAS ratings. The bar chart represents the mean number of ratings received for 'unclear feeling', 'vague feeling', and 'clear feeling' across degrees of visuotactile asynchrony conditions. (D) Perceptual awareness efficiency (M-ratio) model fitting. 10000 iterations of the three Markov Chain Monte Carlo (MCMC) chains per parameter $\mu_{meta\text{-}d'/d'}$ across all degrees of asynchrony, showing that the sampling behaviour is constant over time and that it converged to a stationary distribution. Error bars represent 95% CI.

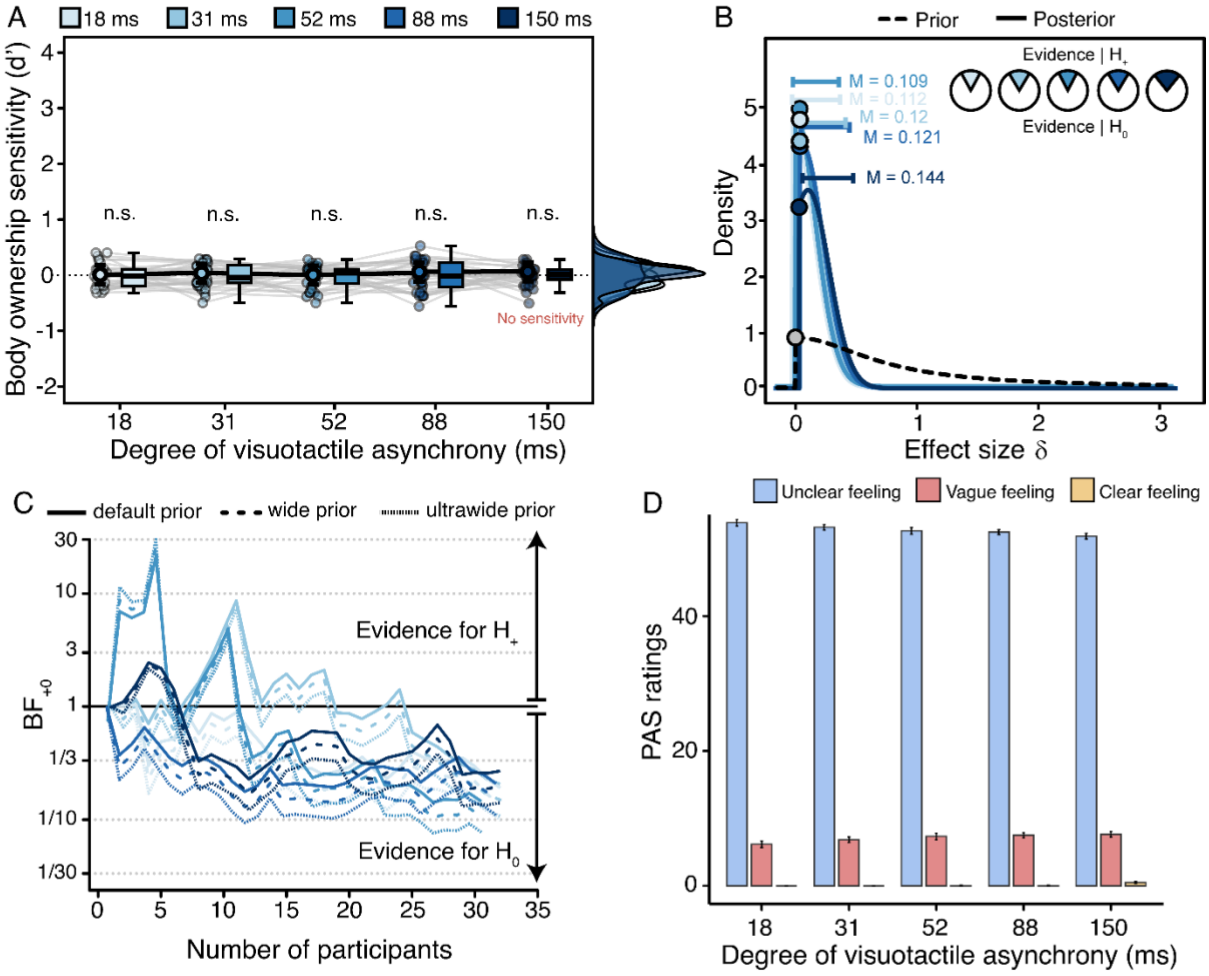

Figure S2. Results of Control Experiment 1: (A-C) Body ownership sensitivity. (A) Individual one-sample t-tests against zero. Body ownership sensitivity did not depart from chance performance with any degree of stimulation asynchrony. (B-C) Bayesian one-sample t tests against zero (one-tailed): (B) Prior and posterior distributions. Bayes factors provided substantial support for the null hypothesis model (i.e., body ownership d' > 0) of all degrees of visuotactile asynchrony. Estimated population effect sizes, medians, and 95% central credible intervals for each asynchrony condition are depicted. (C) Sequential analysis with robustness assessment. The evidence for the null hypothesis model is very stable across different prior distributions, all ranging from 1 to 10 (all r ≤ 1). (D) PAS ratings. The bar chart represents the mean number of ratings received for 'unclear feeling', 'vague feeling', and 'clear feeling' reports across degrees of visuotactile asynchrony conditions. PAS ratings were disproportionately more frequent than for "vague feeling" and "unclear feeling", further supporting that turning the rubber hands in 90 degrees abolishes the RHI. Error bars represent 95% CI.

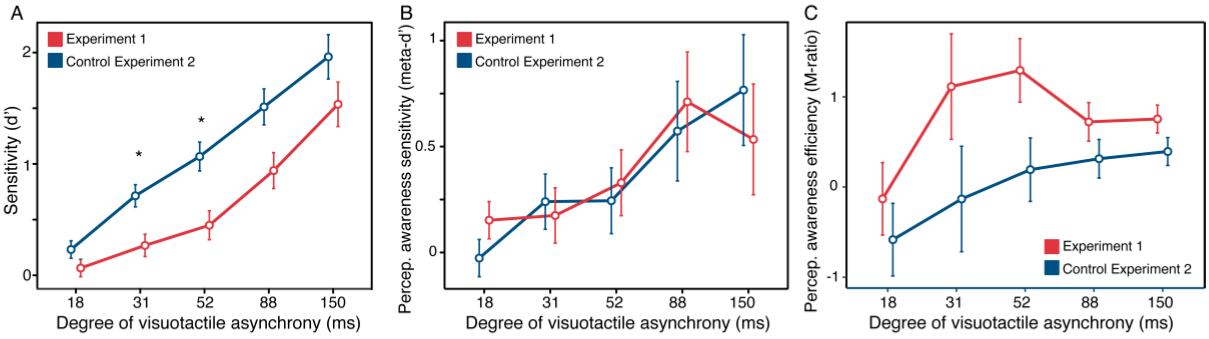

Figure S3. Comparison between Experiment 1 and Control Experiment 2: (A) Body ownership sensitivity vs. visuotactile simultaneity sensitivity. A mixed repeated-measures ANOVA revealed a main effect of degree of asynchrony ($F_{(2.23, 138.31)} = 84.47, p < .001, \eta p^2 = 0.58$), and a significant difference between body ownership sensitivity (Experiment 1) and visuotactile simultaneity sensitivity (Control Experiment 2), ($F_{(1, 62)} = 8.1, p = .006, \eta p^2 = 0.116$). No interaction was observed ($F_{(4, 248)} = 1.66, p = .16, \eta p^2 = 0.26$). (B) Perceptual awareness sensitivity. A mixed repeated-measures ANOVA showed a main effect of degree of asynchrony ($F_{(2.53, 156.76)} = 7.961, p < .001, \eta p^2 = 0.114$), but no difference between Experiment 1 and Control Experiment 2 ($F_{(1, 62)} = 0.011, p = .918, \eta p^2 = 0$). (C) Perceptual awareness efficiency. A mixed repeated-measures ANOVA revealed a main effect of degree of asynchrony ($F_{(2.45, 151.95)} = 3.25, p = .032, \eta p^2 = 0.05$), and crucially, a significant difference between Experiment 1 and Control Experiment 2 ($F_{(1, 62)} = 5.33, p = .024, \eta p^2 = 0.079$), favouring Experiment 1. To enable between-subject comparison, all analyses were conducted on type-1 and type-2 SDT measures obtained using the MLE method. Error bars represent SEM. Asterisks denote exploratory significant differences (*p < .05).

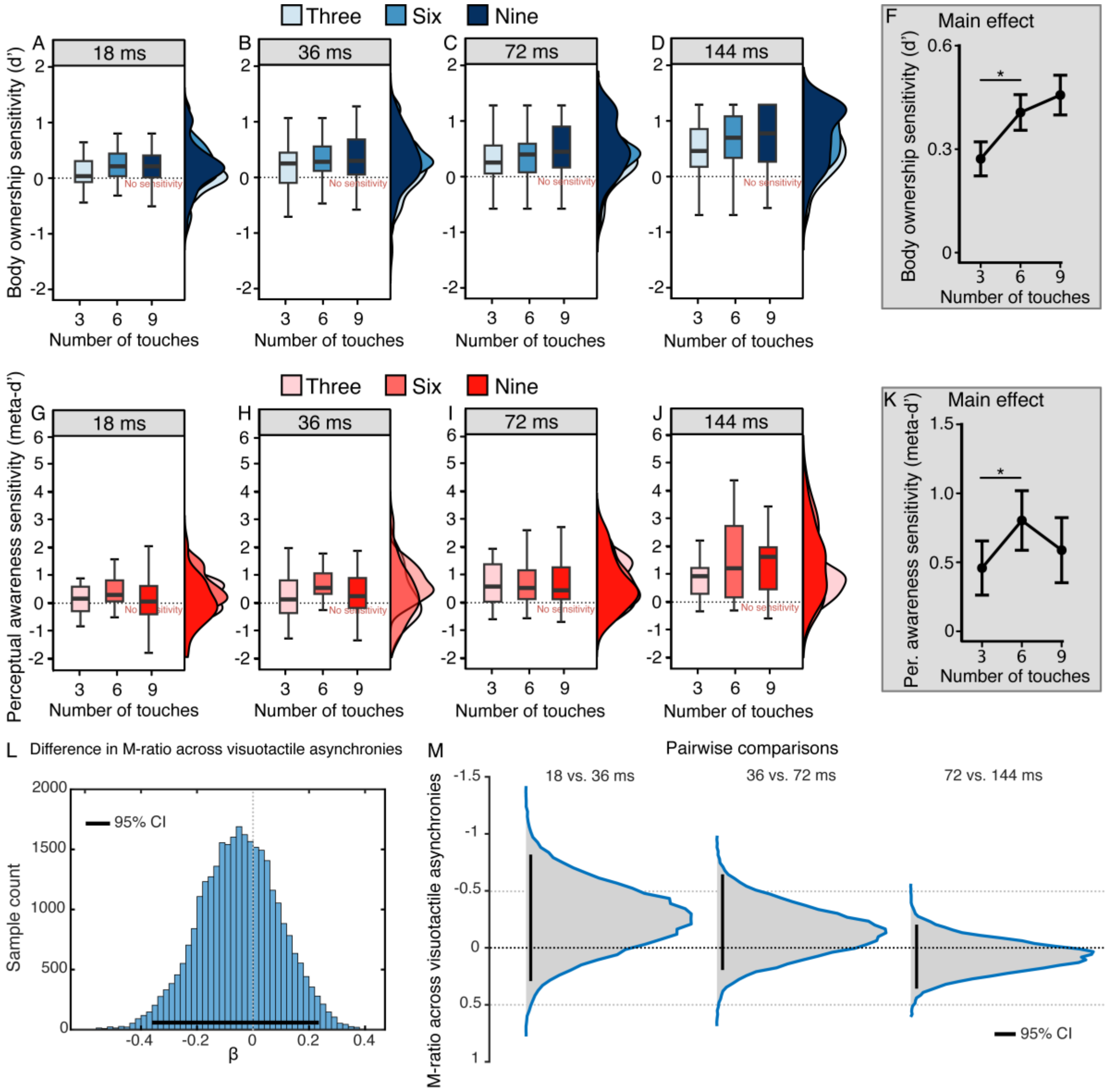

Figure S4. Supplementary results of Experiment 2: Psychophysical measures of body ownership and perceptual awareness sensitivity. (A-F) Body ownership sensitivity (d'): (A-D) Sensitivity to body ownership increases with a greater number of touches and higher degrees of visuotactile asynchrony. (F) The main effect of the number of touches shows that the ability to discriminate visuotactile signals, related to the sense of body ownership, improves with increasing visuotactile evidence. This increase was significant between three and six touches. (G-K) Perceptual awareness sensitivity (meta-d'): (G-J) Perceptual awareness sensitivity also increases with more touches and greater visuotactile asynchrony. (K) The main effect of the number of touches indicates that the subjective experience's ability to predict body ownership sensitivity performance improves with increasing visuotactile evidence, with a significant increase observed between three and six touches. Bayesian hierarchical estimation of perceptual awareness efficiency: (L-M) Perceptual awareness efficiency across degrees of visuotactile asynchrony: (L) Estimation of differences in M-ratio between different degrees of asynchrony suggests no difference between degrees of asynchrony. Results indicate lack of a difference (in log units) between condition posteriors. (M) Pairwise comparisons of posterior distributions between degrees of asynchrony show no difference in perceptual awareness efficiency between different asynchronies. Histograms represent posterior densities of M-ratio for all conditions. Error bars represent 95% CI. Asterisks indicate significant differences (*p < .05).

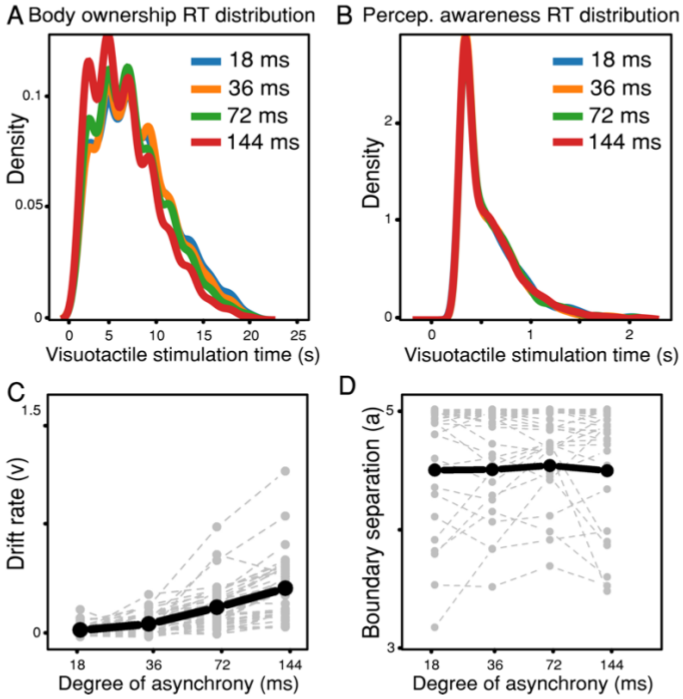

Figure S5. Supplementary results of Experiment 3: (A-B) Response time distributions: (A) Body ownership discrimination response-time distributions across visuotactile stimulation time. A one-way ANOVA did not find an effect of degree of asynchrony ($F_{(3, 120)} = 0.646, p = .587$). (B) Perceptual awareness report response-time distributions across post-decision time. A one-way ANOVA did not find an effect of degree of asynchrony ($F_{(3, 176)} = 1, p = .394$). (C-D): DDM parameters estimated using the v-ratio implementation: (C) Drift rate, which reflects speed of evidence accumulation, increased with increasing degrees of visuotactile asynchrony, thus showing equivalent results to those of the HDDM model (see Fig. 4B). A one-way ANOVA found a main effect of degree of asynchrony ($F_{(3, 156)} = 34.79, p < .001$), and post-hoc comparisons using Tukey HSD for multiple comparisons found differences between all pairs of asynchronies (all p < 0.002), except for the comparison between 18 and 36 ms (p = 0.52). (D) Boundary separation as estimated by the v-ratio implementation did not show differences between degrees of visuotactile asynchrony ($F_{(3, 156)} = 0.02, p = .9$).

Table S1. Rubber hand illusion questionnaire

**Q1.** *It seemed as if I were feeling the touch in the location where I saw the rubber hand touched.*
Q2. *It seemed as though the touch I felt was caused by the stick touching the rubber hand*
Q3. *I felt as if the rubber hand were my hand*
Q4. *It felt as if my (real) hand were drifting towards up (towards the rubber hand).*
Q5. *It seemed as if I might have more than one right hand or arm.*
Q6. *It seemed as if the touch I was feeling came from somewhere between my own hand and the rubber hand*
Q7. *It felt as if my (real) hand was turning 'rubbery'.*
Q8. *It appeared (visually) as if the rubber hand were drifting towards my hand.*
Q9. *The rubber hand began to resemble my own (real) hand, in terms of shape, skin tone, freckles or some other visual feature.*

Note. Each item must be answered using a -3 to +3 Likert scale.

Instructions: Rate the items according to what you felt (-3: I completely disagree; +3: I completely agree).

Table S2. Means and standard deviations for d', meta-d', and M-ratio values of Experiment 1

Asynchrony	d'		Meta-d'		M-ratio	
	M	*SD*	*M*	*SD*	*M*	*SD*
18	0.065	0.365	-0.026	0.433	-0.518	2.194
31	0.268	0.317	0.240	0.481	0.365	3.086
52	0.449	0.353	0.244	0.604	0.829	2.33
88	0.941	0.559	0.573	0.587	0.633	0.639
150	1.535	0.871	0.766	0.930	0.638	0.775

Note. Values were obtained using the MLE method of type-2 SDT analysis. Given the inherent noise in MLE-based M-ratio estimates and the limited number of trials, we used a Bayesian Hierarchical model to test our main M-ratio hypotheses (see **Fig. 2G-H**).

References

1. M. Chancel, B. Hasenack, H. H. Ehrsson, Integration of predictions and afferent signals in body ownership. *Cognition* **212**, 104722 (2021).

2. M. Chancel, H. H. Ehrsson, Which hand is mine? Discriminating body ownership perception in a two-alternative forced-choice task. *Atten Percept Psychophys* **82**, 4058–4083 (2020).

3. H. H. Ehrsson, C. Spence, R. E. Passingham, That's My Hand! Activity in Premotor Cortex Reflects Feeling of Ownership of a Limb. *Science* **305**, 875–877 (2004).

4. R. C. Lanfranco, M. Chancel, H. H. Ehrsson, Quantifying body ownership information processing and perceptual bias in the rubber hand illusion. *Cognition* **238**, 105491 (2023).

5. R. C. Lanfranco, M. Chancel, H. H. Ehrsson, Texture congruence modulates perceptual bias but not sensitivity to visuotactile stimulation during the rubber hand illusion. *Cogn Affect Behav Neurosci* (2024). https://doi.org/10.3758/s13415-024-01155-2.

6. D. M. Lloyd, Spatial limits on referred touch to an alien limb may reflect boundaries of visuo-tactile peripersonal space surrounding the hand. *Brain and Cognition* **64**, 104–109 (2007).

7. H. Nitta, H. Tomita, Y. Zhang, X. Zhou, Y. Yamada, Disgust and the rubber hand illusion: a registered replication report of Jalal, Krishnakumar, and Ramachandran (2015). *Cognitive Research: Principles and Implications* **3**, 15 (2018).

8. M. Tsakiris, M. D. Hesse, C. Boy, P. Haggard, G. R. Fink, Neural Signatures of Body Ownership: A Sensory Network for Bodily Self-Consciousness. *Cerebral Cortex* **17**, 2235–2244 (2007).

9. A. Wold, J. Limanowski, H. Walter, F. Blankenburg, Proprioceptive drift in the rubber hand illusion is intensified following 1 Hz TMS of the left EBA. *Frontiers in Human Neuroscience* **8**, 390 (2014).

10. H. H. Ehrsson, "Chapter 15: Bodily illusions" in *The Routledge Handbook of Bodily Awareness*, (Routledge, 2022), pp. 201–229.

11. M. Botvinick, J. Cohen, Rubber hands 'feel' touch that eyes see. *Nature* **391**, 756–756 (1998).

12. C. Fan, S. Coppi, H. H. Ehrsson, The supernumerary rubber hand illusion revisited: Perceived duplication of limbs and visuotactile events. *J Exp Psychol Hum Percept Perform* **47**, 810–829 (2021).

13. M. Overgaard, K. Sandberg, The Perceptual Awareness Scale—recent controversies and debates. *Neuroscience of Consciousness* **2021**, niab044 (2021).

14. N. A. Macmillan, C. D. Creelman, *Detection Theory: A User's Guide*, 2 edition (Lawrence Erlbaum, 2004).

15. T. D. Wickens, *Elementary Signal Detection Theory* (Oxford University Press, 2001).

16. B. Maniscalco, H. Lau, A signal detection theoretic approach for estimating metacognitive sensitivity from confidence ratings. *Consciousness and Cognition* **21**, 422–430 (2012).

17. B. Maniscalco, H. Lau, "Signal detection theory analysis of type 1 and type 2 data: Meta-d', response-specific meta-d', and the unequal variance SDT model" in *The Cognitive Neuroscience of Metacognition*, (Springer-Verlag Publishing, 2014), pp. 25–66.

18. S. M. Fleming, HMeta-d: hierarchical Bayesian estimation of metacognitive efficiency from confidence ratings. *Neuroscience of Consciousness* **2017**, nix007 (2017).

19. S. M. Fleming, H. C. Lau, How to measure metacognition. *Front. Hum. Neurosci.* **8** (2014).

20. J. Kruschke, *Doing Bayesian Data Analysis: A Tutorial with R, JAGS, and Stan*, 2nd edition (Academic Press, 2014).

21. M. Ide, The Effect of "Anatomical Plausibility" of Hand Angle on the Rubber-Hand Illusion. *Perception* **42**, 103–111 (2013).

22. M. Tsakiris, P. Haggard, The rubber hand illusion revisited: visuotactile integration and self-attribution. *J Exp Psychol Hum Percept Perform* **31**, 80–91 (2005).

23. M. Tsakiris, L. Carpenter, D. James, A. Fotopoulou, Hands only illusion: multisensory integration elicits sense of ownership for body parts but not for non-corporeal objects. *Exp Brain Res* **204**, 343–352 (2010).

24. G. Finotti, S. Garofalo, M. Costantini, D. R. Proffitt, Temporal dynamics of the Rubber Hand Illusion. *Sci Rep* **13**, 7526 (2023).

25. R. Ratcliff, Modeling response signal and response time data. *Cognitive Psychology* **53**, 195–237 (2006).

26. R. Ratcliff, P. L. Smith, S. D. Brown, G. McKoon, Diffusion Decision Model: Current Issues and History. *Trends in Cognitive Sciences* **20**, 260–281 (2016).

27. T. Wiecki, I. Sofer, M. Frank, HDDM: Hierarchical Bayesian estimation of the Drift-Diffusion Model in Python. *Frontiers in Neuroinformatics* **7** (2013).

28. W. Pan, *et al.*, dockerHDDM: A user-friendly environment for Bayesian Hierarchical Drift-Diffusion Modeling. [Preprint] (2022). Available at: https://osf.io/6uzga [Accessed 1 October 2024].

29. K. Desender, L. Vermeylen, T. Verguts, Dynamic influences on static measures of metacognition. *Nat Commun* **13**, 4208 (2022).

30. R Core Team, R: A language and environment for statistical computing. (2003). Deposited 2003.

31. JASP Team, JASP. (2023). Deposited 2023.
